# The intratumoral microbiome in colorectal cancer: origins, microenvironmental interactions, and new horizons in precision medicine

**DOI:** 10.3389/fimmu.2026.1795736

**Published:** 2026-02-27

**Authors:** Xuemei Li, Qian Wang, Qiang Yuan, Li Wang

**Affiliations:** 1 Department of Clinical Medicine, Chengdu Medical College, Chengdu, Sichuan, China; 2 Department of Gastroenterology, First Affiliated Hospital of Chengdu Medical College, Chengdu, Sichuan, China

**Keywords:** biomarkers, colorectal cancer, *Fusobacterium nucleatum*, immunotherapy, intratumoral microbiome, tumor microenvironment

## Abstract

As a key functional component of the tumor microenvironment (TME), the intratumoral microbiome in colorectal cancer (CRC) has revolutionized the traditional paradigm of the “sterile tumor.” Far from being mere “bystanders,” these intratumoral microbes act as key drivers deeply implicated in remodeling the TME, influencing tumor progression, and determining therapeutic responses, thus necessitating a comprehensive synthesis of their complex biological characteristics and potential for clinical translation. Therefore, this review systematically summarizes the potential origins, community characteristics, and anatomical heterogeneity of the intratumoral microbiome. It further explores the precise mechanisms driving tumor progression, including the induction of genomic instability, metabolic reprogramming, epigenetic regulation, and immune microenvironment remodeling. We highlight the clinical utility of intratumoral microbes in CRC diagnosis, prognosis, and therapeutic prediction, while also introducing novel intervention strategies based on nanomedicine, engineered probiotics, and phage therapy. Finally, critical challenges such as contamination control in low-biomass samples, sampling heterogeneity, and the delineation of causality are scrutinized, aiming to provide new perspectives for the development of microbiome-guided precision medicine in CRC.

## 1 Introduction

Colorectal cancer (CRC) is a malignancy characterized by high global incidence and mortality rates (1). Its pathogenesis involves complex interactions among the host genome, environmental factors, and the tumor microenvironment (TME) (2). Historically, microbiome research has primarily focused on the microbiota within the gut lumen. However, recent advancements in high-throughput sequencing and spatial multi-omics technologies have revealed a critical fact: tumor tissues are not sterile. Instead, they are colonized by an “intratumoral microbiome” that possesses specific spatial distributions and metabolic activities (3, 4). Studies indicate that the intratumoral microbiome differs significantly in composition from the luminal microbial community. Moreover, it exhibits distinct tumor-enrichment characteristics (5).

The intratumoral microbiome is not a result of random colonization or “contamination”; rather, it represents a highly heterogeneous ecosystem. During the adenoma-to-carcinoma sequence, the composition and abundance of this microbiome undergo dynamic reorganization. Furthermore, these microbes are deeply involved in the initiation and progression of CRC. They drive these processes by inducing genomic instability, reshaping the immune microenvironment, and activating specific oncogenic pathways (6, 7). For instance, the enrichment of key pathogens, such as *Fusobacterium nucleatum* and enterotoxigenic *Bacteroides fragilis* (ETBF), has been confirmed in tumor tissues. This enrichment is closely associated with poor prognosis and metastasis (8–10). Currently, research focusing on the intratumoral microbiome is experiencing explosive growth. The rapid development of this field marks a paradigm shift in cancer biology. We no longer view tumors merely as aggregations of cells; instead, we redefine them as complex ecosystems composed of host cells and microbes interacting intimately.

This article aims to systematically review the past decade of research regarding the intratumoral microbiome in CRC. We cover its potential origins, characteristics of community distribution, research methodologies, and mechanisms of interaction with the TME. Additionally, we discuss its clinical value and the challenges currently facing this field. Our goal is to provide new perspectives for the development of novel diagnostic biomarkers and microbiome-directed therapeutic strategies for CRC.

## 2 Potential origins and colonization of the intratumoral microbiome in CRC

To gain a deeper understanding of the function and clinical significance of the intratumoral microbiome, a critical question must be addressed: where do these microbes originate, and via which pathways do they colonize the tumor? While the exact origin of intratumoral microbes in CRC is still under investigation, current evidence primarily points to two main pathways. The first is endogenous translocation from the gut microbial reservoir into tumor tissues, often occurring against a backdrop of damaged mucosal barriers. The second is the migration of microbes from distal sites, such as the oral cavity, via the circulatory or lymphatic systems, followed by selective colonization within the TME. As the human body’s largest bacterial reservoir, the gut microbiome is widely considered the primary source of microbes within gastrointestinal malignancies (9). Based on the concept of the “gut-tumor microbiota axis,” the gut and tumor sites share more than just anatomical continuity. There is also dynamic communication and functional synergy between their microbial communities (11).

Current hypotheses propose several specific mechanisms. First, impairment of the intestinal mucosal barrier may allow luminal bacteria to breach epithelial defenses and directly colonize neoplastic tissues (6). Second, local inflammation can compromise barrier homeostasis and facilitate the migration of microbes from adjacent tissues into the tumor. This process simultaneously amplifies the production of inflammatory factors, thereby accelerating tumor growth (12). Additionally, hematogenous dissemination is considered a potential source. Microbes may enter the circulation through damaged blood vessels and subsequently accumulate at the tumor site. Some theories suggest that certain bacteria may even migrate to distant organs alongside tumor cells, a process known as “co-metastasis” (6). This concept has been partially substantiated in studies of metastatic CRC. For example, research has been conducted on pseudomyxoma peritonei (PMP), a condition often originating from a ruptured appendiceal tumor that spreads to the peritoneal cavity. In metastatic tumor tissues, researchers identified reproducible core microbial taxa. These taxa likely originate from the rupture and dissemination of the primary site and include specific bacterial groups closely associated with CRC (13). These findings support the hypothesis that intratumoral microbes can transfer from the primary tumor or gut environment to colonize distant tumor tissues, potentially facilitated by physical barrier disruption or cellular carriers.

The “oral-gut” axis plays a crucial role in shaping the intratumoral microbiome in CRC. Studies have revealed that tumor tissues in CRC patients are enriched with bacteria of oral origin. These include *F. nucleatum*, *Porphyromonas gingivalis*, and *Prevotella* species. The homology between these bacteria in oral and intestinal lesions suggests specific colonization pathways. Oral pathogens may colonize the colorectal mucosa via hematogenous dissemination or swallowing through the digestive tract, subsequently inducing or promoting tumorigenesis (14–16). Oral diseases such as periodontitis cause dysbiosis, which may increase the risk of oral pathogen translocation to the gut. These pathogens then participate in CRC pathology by disrupting the intestinal barrier and modulating immune responses (14, 17). This mechanism of cross-site microbial migration not only explains the source of specific intratumoral bacterial communities but also provides a potential biological basis for CRC screening using oral microbial detection.

## 3 Compositional and distribution characteristics of the CRC intratumoral microbiome

**Figure 1:**
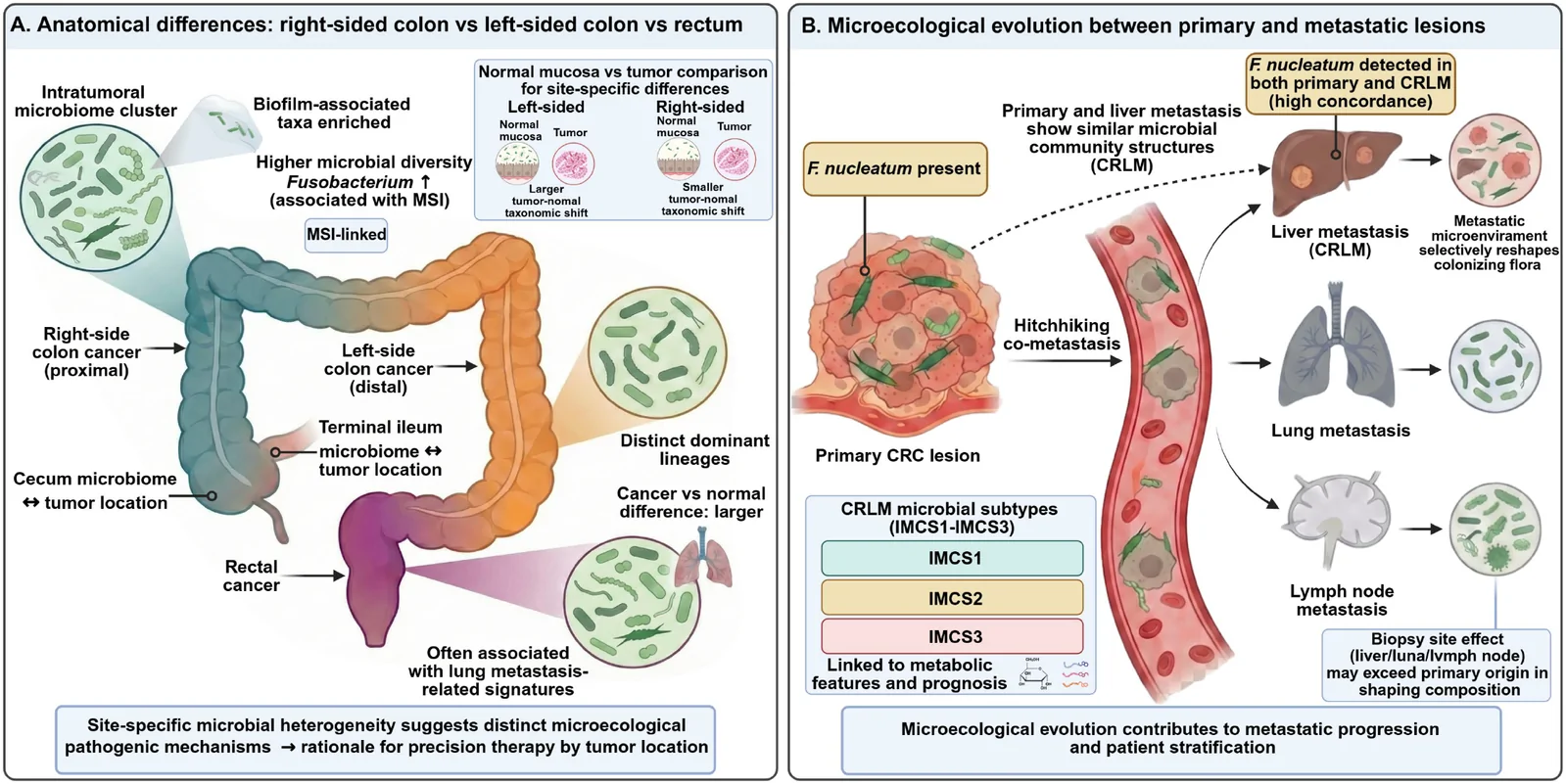
Compositional and distribution characteristics of the intratumoral microbiome in CRC. **(A)** Anatomical differences across CRC sites. Schematic comparison of intratumoral microbial lineages across right-sided (proximal) colon cancer, left-sided (distal) colon cancer, and rectal cancer. Right-sided colon tumors typically exhibit higher microbial diversity and are more prone to enrichment of biofilm-associated taxa; increased abundance of *Fusobacterium* is often associated with microsatellite instability (MSI). Left-sided colon cancer and rectal cancer display distinct dominant microbial lineages, and rectal cancer–associated microbial signatures are frequently linked to lung metastasis. The inset highlights site-dependent magnitudes of the “normal mucosa–tumor” shift, showing a larger tumor–normal difference in left-sided disease but a relatively smaller difference in right-sided disease. In addition, the cecal and terminal ileal microbiomes are associated with tumor location, indicating site-specific heterogeneity of the intratumoral microecology. **(B)** Microecological evolution between primary and metastatic lesions. Intratumoral microbes can accompany tumor cells during metastatic dissemination (“hitchhiking co-metastasis”), resulting in partial similarity of microbial community structures between primary tumors and metastatic lesions, particularly colorectal cancer liver metastasis (CRLM). Using *Fusobacterium nucleatum* as an example, high concordance can be observed between primary CRC and paired CRLM. Meanwhile, the metastatic microenvironment (liver, lung, lymph node) can selectively reshape the colonizing flora. The figure further illustrates that CRLM can be stratified into distinct subtypes based on intratumoral microbial community features (IMCS1–IMCS3; intratumoral microbial community subtypes), which are associated with metabolic characteristics and prognosis, and emphasizes that in some settings the biopsy-site effect may outweigh the primary origin in shaping the microbial composition of metastatic lesions. Created in BioRender. Steven, T. (2026) https://BioRender.com/t4aum9z.

The CRC intratumoral microbiome is spatially heterogeneous rather than uniform, and it is reshaped by anatomical location, local ecological niches, and metastatic dissemination. Site-specific tumors exhibit distinct lineage compositions and different magnitudes of tumor–normal divergence, whereas metastatic progression may involve microbial “hitchhiking” with tumor cells followed by selective filtering by the metastatic microenvironment. This section summarizes these compositional and distributional features, as overviewed in **Figure 1**, while representative studies delineating specific microbial taxa across different clinical contexts are compiled in **Table 1**.

**Table 1:** Summary of studies on the composition and distribution characteristics of the intratumoral microbiome in CRC.

Subtype/anatomical site	Key microbial taxa	Distribution features	Sample size	Country	Method	Reference
Right-sided (RSCC) vs left-sided (LSCC) colon cancer	RSCC was characterized by higher relative abundance of *Haemophilus* and *Veillonella*, whereas LSCC showed higher relative abundance of *Bifidobacterium*, *Akkermansia*, *Roseburia*, and *Ruminococcus*.	Distinct tumor microbiomes by side; Grade 3 tumors enriched in *Fusobacterium* and *Parvimonas* (biofilm-formers)	41	Germany	16S rRNA gene sequencing (tumor biopsies)	Kneis et al., 2023 (22)
Consensus molecular subtypes (CMS) CMS1 vs CMS2 vs CMS3 CRC	CMS1 was characterized by *Fusobacterium* (e.g., *F. hwasookii*, *F. nucleatum*), *Porphyromonas gingivalis*, *Parvimonas micra*, and *Peptostreptococcus stomatis*; CMS2 was characterized by *Selenomonas* and *Prevotella* spp.; CMS3 showed limited/weak microbial subtype associations.	Distinct bacterial species signatures by CMS; oral pathogen enrichment in CMS1 subtype	34	New Zealand	16S rRNA sequencing & tumor RNA‐seq	Purcell et al., 2017 (101)
MSI‐high vs MSI‐low (MSS) CRC	MSI-high CRC was characterized by higher relative abundance of *Dialister* and *Prevotella* (oral-associated genera), with multiple *Fusobacteriaceae* genera also more prevalent in MSI-high tumors.	MSI‐high tumors show higher intratumoral microbial diversity; multiple genera differ by MSI status	451 (TCGA cohort)	USA	Whole‐genome/transcriptome sequencing (TCGA) microbial profiling	Byrd et al., 2023 (102)
Tumor‐associated biofilms in CRC (Indian cohort)	Tumor-associated biofilms were dominated by *Escherichia coli*, *Klebsiella pneumoniae*, and *Bacteroides fragilis*, while *Fusobacterium nucleatum* and *pks*+ *E. coli* were less prevalent in these biofilm-positive tumors.	Tumor tissues show invasive biofilms with distinct dysbiosis vs adjacent mucosa	15 tumors (with 15 matched adjacent)	India	FISH (16S rDNA probes) & confocal microscopy	Kushwaha et al., 2025 (103)
Young‐onset (<50) vs average‐onset (>60) CRC	Young-onset CRC showed higher relative abundance of *Akkermansia* and *Bacteroides*, whereas average-onset CRC was characterized by *Bacillus*, *Staphylococcus*, *Listeria*, *Enterococcus*, *Pseudomonas*, *Fusobacterium*, and *Escherichia/Shigella*.	Young-onset tumors had higher alpha diversity and distinct microbiome composition vs older patients	136 vs 140	USA	16S rRNA gene sequencing	Barot et al., 2024 (38)
CRC with vs without liver metastasis (CRLM)	CRC with liver metastasis was characterized by *Odoribacter*, *Leptothrix*, *Clavibacter*, and *Caulobacter*, while non-metastatic CRC was characterized by *Agrobacterium*, *Fusobacterium*, *Methylobacterium*, and *Faecalibacterium*.	Distinct intratumoral microbiomes in metastatic vs non-metastatic CRC; *Fusobacterium* notably depleted in metastases	44 vs 85	China	Targeted 16S rRNA sequencing (5R method)	Yan et al., 2025 (25)
Primary CRC tumor vs metastasis (metastasis risk)	Metastasizing primary tumors were characterized by enterotoxigenic *Bacteroides fragilis* alongside lower relative abundance of *Fusobacterium nucleatum*; metastatic lesions were characterized by *Enterobacteriaceae* (e.g., *Escherichia coli*).	Primary tumors had higher microbial diversity; specific microbial markers (3-species clique) predict metastasis risk	900 (primary tumors, ORIEN)	USA	RNA-seq microbiome profiling; 16S rDNA qPCR	Parajuli et al., 2025 (104)
Adenoma (A) vs tumor (T) vs para-carcinoma (P) tissues (multi-kingdom, tissue-resident microbiome)	Across adenoma-to-carcinoma progression, *Fusobacterium nucleatum*, *Bacteroides fragilis*, *Parvimonas micra*, and *Prevotella intermedia* were repeatedly implicated, with tumor tissue showing greater representation of *Bacteroides*/*Klebsiella*; multi-kingdom taxa (bacteria, fungi, archaea, viruses) were detected.	Distinct dysbiosis across A→T with reduced α-diversity; tumor and para-carcinoma show similar overall bacterial/fungal composition, while archaea remain relatively stable; fungal colonization supported by FISH	62 (adenoma) vs 62 (tumor)	China	Deep shotgun metagenomics + proteomics + FISH	Wu et al., 2025 (3)
Unsupervised microbiome clusters (two main clusters) based on phylum-level composition	Dominant phyla: Proteobacteria (43.5%), Firmicutes (25.3%), Actinobacteria (23.0%), Bacteroidetes (5.1%); cluster1 enriched in Proteobacteria & Bacteroidetes, cluster2 in Firmicutes & Actinobacteria	Two clusters had significantly different prognoses (cluster1 with higher Proteobacteria & Bacteroidetes showed poorer survival vs cluster2)	533 (TCGA samples)	China	Metagenomic analysis of TCGA data	Xu et al., 2023 (105)
Tumor vs adjacent normal tissue	Tumor tissue showed greater representation of *Fusobacterium* (including *F. nucleatum* and *F. animalis/polymorphum*), *Leptotrichia*, and *Streptococcus*, while *Bacteroides* and *Corynebacterium* were less represented in tumors compared with adjacent normal tissue.	Some location differences: rectal and sigmoid tumors tended to have higher Fusobacterium than colon tumors (colon tumors had relatively less *F. nucleatum/polymorphum*, *Staphylococcus*, *Streptococcus*)	65 (paired tumor vs normal mucosa)	Spain	16S rRNA gene sequencing	González et al., 2024 (106)

RSCC/LSCC, right-sided/left-sided colon cancer; CMS, consensus molecular subtypes (CMS1–CMS4); MSI/MSS, microsatellite instability/microsatellite stable; CRLM, colorectal cancer liver metastasis; FISH, fluorescence *in situ* hybridization; WGS, whole-genome sequencing; qPCR, quantitative polymerase chain reaction; TCGA, The Cancer Genome Atlas; ORIEN, Oncology Research Information Exchange Network.

### 3.1 Anatomical differences: a comparison of microbial lineages in left-sided colon, right-sided colon, and rectal cancers

The heterogeneity of CRC is largely reflected in the differences in intratumoral microbial communities across distinct anatomical sites. Studies indicate that proximal (right-sided) colon cancer possesses a distinct microbial lineage compared to distal (left-sided) colon cancer and rectal cancer. Right-sided colon cancer typically exhibits higher microbial diversity. It is also more prone to enriching bacterial taxa associated with biofilm formation (18, 19). For example, research has found that the abundance of *Fusobacterium* is significantly higher in right-sided tumors than in left-sided ones. This abundance is closely related to microsatellite instability (MSI) status (18, 20). Conversely, left-sided colon cancer and rectal cancer show different dominant bacterial groups, which are often associated with lung metastasis (21). Meanwhile, the patterns of difference between normal mucosa and cancerous tissue vary by anatomical site. In patients with left-sided colon cancer, the taxonomic difference between cancerous and non-cancerous tissues is substantial. However, this difference is relatively smaller in patients with right-sided colon cancer (19). Furthermore, microbiomes in the cecum and terminal ileum exhibit specific associations related to tumor location (22). This heterogeneity in microbial distribution based on anatomical sites suggests that tumors in different locations may possess unique microecological pathogenic mechanisms. This provides a theoretical basis for site-specific precision therapy.

### 3.2 Microecological evolution between primary and metastatic lesions

Intratumoral microbes are not confined to primary tumors; they also migrate to distant organs accompanying the metastasis of tumor cells. Studies on colorectal cancer liver metastasis (CRLM) have found significant similarity in microbial community structures between primary and liver metastatic lesions. This suggests that bacteria may “hitchhike” with tumor cells during metastasis (8, 23). For instance, *F. nucleatum* has been detected in both primary CRC and its liver metastases. Its presence in metastatic lesions is highly consistent with that in the primary tumor. Even in germ-free mouse models, tail vein injection of this bacterium leads to its specific colonization at tumor sites (23). However, the microenvironment of the metastatic site also selectively reshapes the colonizing flora. Some studies note that while the microbial diversity of metastases is influenced by the primary tumor type, the biopsy site (e.g., liver, lung, or lymph nodes) can sometimes influence microbial composition more than the primary tumor source does (24). Additionally, patients with liver metastases can be classified into different subtypes based on intratumoral microbial community characteristics (25). These subtypes are closely associated with metabolic features and prognosis. This reveals the critical role of microecological evolution in metastatic progression.

## 4 Key research methods and technological advances for profiling the intratumoral microbiome in CRC

As our understanding of intratumoral microbial functions deepens, research methodologies have undergone a profound evolution from traditional culture techniques to multi-omics joint analysis. This evolution aims to overcome technical bottlenecks such as low sample biomass, high host background interference, and ambiguous spatial localization. 16S rRNA gene sequencing remains the cornerstone for delineating microbial community composition, yet it is limited by low resolution. Currently, the application of metagenomics allows researchers to achieve precision at the species or even strain level and to mine potential functional genes. For instance, whole-genome sequencing technology has successfully identified novel strains in colorectal tumor tissues, such as *Klebsiella michiganensis*. This has revealed their unique virulence factors and antibiotic resistance genes (26). More importantly, integrated multi-omics analysis is becoming a mainstream trend. By integrating metagenomic, proteomic, and transcriptomic data, researchers can not only identify microbial species but also dissect dynamic host-microbe interaction networks at the protein level. This facilitates the construction of classifiers with higher diagnostic efficacy (3, 27). The introduction of artificial intelligence and machine learning algorithms has further enhanced the ability to extract key features from massive microbial datasets. For example, key differentially abundant microbial features can be screened using linear discriminant analysis (LDA), or random forest models can be built to predict responses to chemoradiotherapy (28, 29).

The improvement of spatial resolution represents the forefront of current technological evolution. Traditional bulk tissue sequencing obscures the spatial distribution characteristics of microbes. Modern studies utilize spatial transcriptomics combined with *in situ* hybridization techniques to precisely map microbial localization within the TME. Researchers have successfully elucidated the spatial associations between bacterial communities and tumor cell proliferative activity, vascularization levels, and immune status (30). Modified versions of single-cell RNA sequencing (scRNA-seq) technologies, such as INVADEseq and the CSI-Microbes computational pipeline, have made it possible to detect intracellular bacteria at single-cell resolution. These technologies reveal that tumor cells infected by bacteria upregulate antigen presentation pathways, while myeloid cells that phagocytose bacteria serve as the primary source of bacterial RNA (30, 31). These high-precision technical tools are reconstructing our understanding of the intratumoral microbial ecosystem in unprecedented dimensions.

Establishing appropriate preclinical model systems is particularly urgent to verify causal relationships and dissect molecular mechanisms under controlled conditions. Traditional cell culture cannot simulate the complex TME, but the emergence of organoid technology has filled this gap. Intestinal organoids can recapitulate the structural and functional characteristics of the intestinal epithelium. They provide high-quality *in vitro* models for studying host-microbe-metabolite interactions and show great potential in exploring the pathogenesis of early-onset CRC (EO-CRC) (32, 33). Regarding *in vivo* models, germ-free mice and gnotobiotic mouse models remain the gold standard for verifying the carcinogenic or tumor-suppressive functions of specific bacteria (34). By colonizing these models with patient-derived microbiota or specific engineered bacteria, researchers can simulate the initiation and progression of human CRC and evaluate the efficacy of microbiome modulation strategies. This is decisive for distinguishing whether microbes are the “cause” or the “consequence” of CRC.

## 5 Composition of the core intratumoral microbiota and host–microbe interaction mechanisms

**Figure 2:**
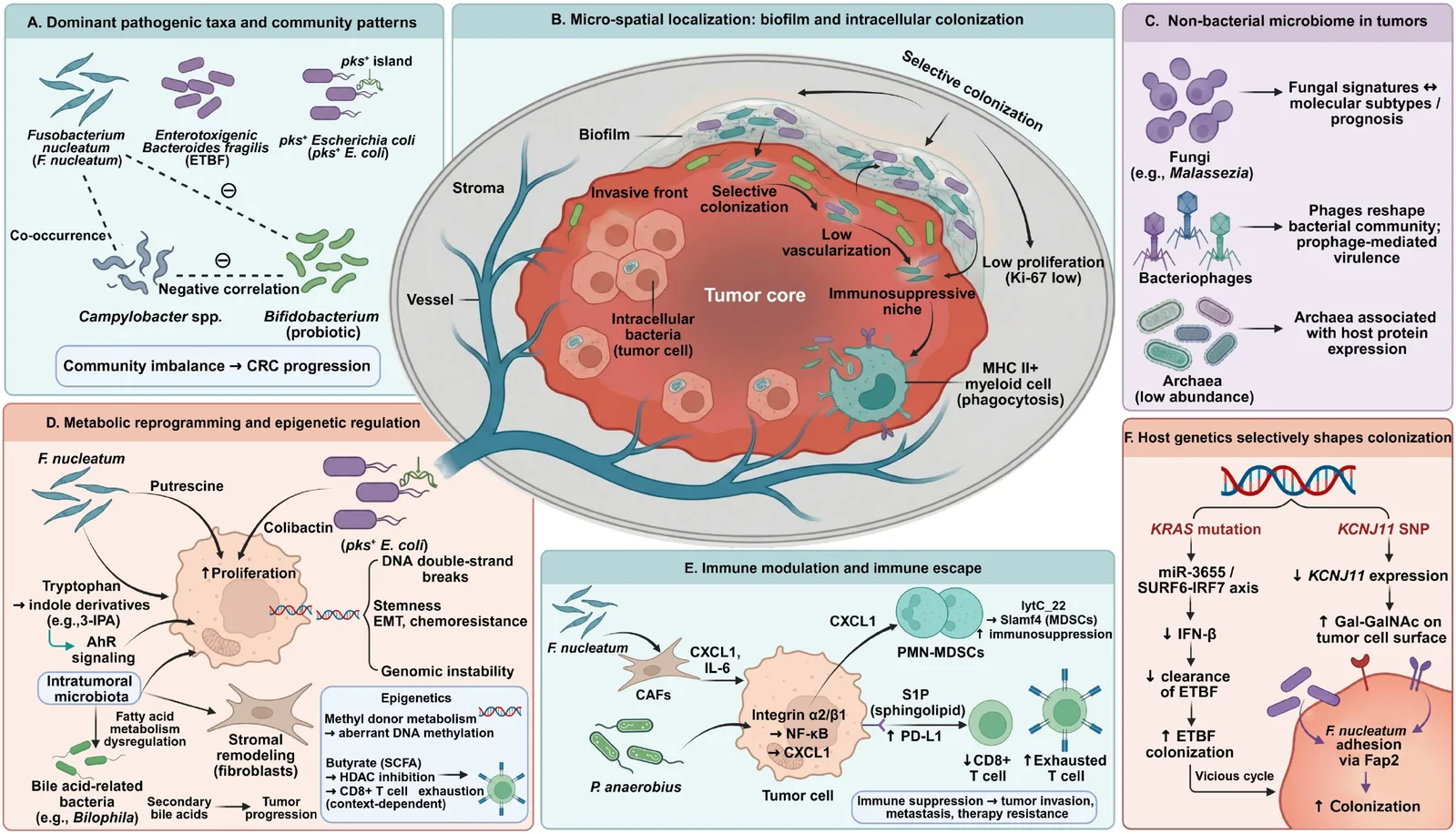
Core intratumoral microbiota and host–microbe interaction mechanisms in CRC. **(A)** Dominant pathogenic taxa and community patterns. *Fusobacterium nucleatum* (*F. nucleatum*), enterotoxigenic *Bacteroides fragilis* (ETBF), and *pks* island–harboring *Escherichia coli* (*pks*+ E. coli) represent core pro-tumorigenic taxa in CRC. *F. nucleatum* frequently co-occurs with other pathogens (e.g., *Campylobacter* spp.) and shows negative correlation with probiotics (e.g., *Bifidobacterium*), contributing to community imbalance and CRC progression. **(B)** Micro-spatial localization: biofilm and intracellular colonization. Intratumoral microbes display selective colonization, including biofilm formation at the invasive front and enrichment within microniches characterized by low vascularization, immunosuppressive features, and low proliferation (Ki-67 low). Microbes can localize intracellularly in tumor cells and be phagocytosed by major histocompatibility complex class II–positive (MHC II+) myeloid cells. **(C)** Non-bacterial microbiome in tumors. Fungi (e.g., *Malassezia*), bacteriophages, and archaea (low abundance) may associate with molecular subtypes/prognosis, reshape bacterial community structure, and contribute to host–microbe interactions. **(D)** Metabolic reprogramming and epigenetic regulation. *F. nucleatum*-derived metabolites (e.g., putrescine) promote tumor proliferation, while tryptophan-to-indole derivatives (e.g., 3-indolepropionic acid, 3-IPA) activate aryl hydrocarbon receptor (AhR) signaling to enhance stemness, epithelial–mesenchymal transition (EMT), and chemoresistance. *pks*+ **(E)** coli produces colibactin, inducing DNA double-strand breaks and genomic instability. Broader intratumoral microbiota perturbations drive fatty acid metabolism dysregulation and stromal remodeling (fibroblasts), and bile acid–related bacteria (e.g., *Bilophila*) contribute to secondary bile acids that support tumor progression. Epigenetically, altered methyl donor metabolism promotes aberrant DNA methylation, whereas butyrate, a short-chain fatty acid (SCFA), via histone deacetylase (HDAC) inhibition can induce context-dependent CD8+ T cell exhaustion. **(E)** Immune modulation and immune escape. *F. nucleatum* activates cancer-associated fibroblasts (CAFs) to secrete chemokine (C-X-C motif) ligand 1 (CXCL1) and interleukin-6 (IL-6), facilitating invasion and metastasis. *Peptostreptococcus anaerobius* triggers integrin α2/β1–NF-κB signaling in tumor cells to induce CXCL1, recruiting polymorphonuclear myeloid-derived suppressor cells (PMN-MDSCs). The bacterial factor lytC_22 enhances MDSC immunosuppression via signaling lymphocytic activation molecule family member 4 (SLAMF4) on MDSCs. Microbial sphingolipid sphingosine-1-phosphate (S1P) upregulates programmed death-ligand 1 (PD-L1) on tumor cells, restricting effector T-cell expansion and increasing exhausted T cells, collectively driving immune suppression and therapy resistance. **(F)** Host genetics selectively shapes colonization. *KRAS* mutation–associated miR-3655/SURF6–interferon regulatory factor 7 (IRF7) signaling reduces interferon beta (IFN-β), impairing ETBF clearance and increasing ETBF colonization in a vicious cycle. *KCNJ11* single nucleotide polymorphism (SNP)–linked downregulation of *KCNJ11* increases galactose–N-acetylgalactosamine (Gal–GalNAc) on tumor cells, promoting *F. nucleatum* adhesion via Fap2 and enhancing colonization. Created in BioRender. Steven, T. (2026) https://BioRender.com/bjwm3mm.

The CRC intratumoral microbiome comprises a limited set of core pathogenic taxa together with non-bacterial members, and it promotes tumor progression through coordinated axes including metabolic reprogramming, epigenetic modulation, immune microenvironment remodeling, and host genetic selection. Rather than acting in isolation, these microbes often form community-level networks via co-occurrence/co-exclusion and exhibit spatially organized colonization with functional specialization. **Figure 2** provides an overview of the core intratumoral taxa in colorectal cancer and their major host–microbe interaction mechanisms.

### 5.1 Dominant pathogenic taxa: enrichment patterns of *F. nucleatum*, enterotoxigenic *Bacteroides fragilis*, and *Escherichia coli*

In the complex microecological network of CRC, several core pathogenic bacterial groups have been repeatedly confirmed to exert significant pro-tumorigenic effects. *F. nucleatum* is the most extensively studied “star bacterium.” *F. nucleatum* not only colonizes tissues itself but often co-exists with other pathogens, such as *Campylobacter* species, to form a pro-tumorigenic microecological network (35). ETBF represents another key pathogen. Its secreted toxins have been confirmed to induce an invasive phenotype in tumor cells. Furthermore, it drives tumor transformation toward the mesenchymal subtype (consensus molecular subtype 4, CMS4), which is associated with a poorer prognosis (36). Additionally, *E. coli* carrying the *pks* genomic island (*pks*+ *E. coli*) produces the genotoxin colibactin. This toxin causes host DNA double-strand breaks and induces genomic instability, thereby directly promoting tumorigenesis (37). These dominant microbial groups do not exist in isolation. They often exhibit patterns of co-exclusion or co-occurrence within tumor tissues. For instance, some studies have observed a negative correlation between *F. nucleatum* and probiotics such as *Bifidobacterium* (38). This imbalance in community structure acts as a significant driving force for CRC progression.

### 5.2 Non-bacterial microbiome: potential distribution and roles of fungi, viruses, and archaea in tumors

Although bacteria are the current focus of research, the roles of the non-bacterial microbiome (fungi, viruses, and archaea) within CRC tumors are gradually emerging. Fungi, in particular, play a role in the TME that cannot be ignored. For example, studies have identified an enrichment of *Malassezia* in hepatocellular carcinoma. This fungus promotes tumor progression by downregulating bile acid synthesis and modulating the TME. This mechanism may hold potential reference value for CRC liver metastasis (39). In CRC, specific fungal signatures have also been associated with molecular subtypes and prognosis (3). Furthermore, bacteriophages, as bacterial viruses, may indirectly influence tumor progression by regulating bacterial community structure. Alternatively, they may integrate directly into bacterial genomes to propagate virulence factors, as seen with specific prophage regions discovered in CRC-associated bacteria (26). Although archaea are present in lower abundance, they have been confirmed in CRC tissues. Their association with specific host protein expression suggests they may play underappreciated roles in host-microbe interactions (3).

### 5.3 Host-microbe interaction: carcinogenic mechanisms based on metabolic reprogramming and epigenetic regulation

Intratumoral microbes profoundly influence tumor cell metabolic reprogramming and gene expression by secreting metabolites and modulating host epigenetic modifications. *Staphylococcus sciuri* converts L-lysine to Nα-acetyl-L-lysine via its GCN5-related N-acetyltransferase. This subsequently activates oxidative stress-related JNK and JAK/STAT signaling pathways through the host Loxl2/H2O2 axis, promoting intestinal stem cell division and tumor growth (40). Alterations in fatty acid metabolism serve as a critical bridge connecting microbes and the TME. Research indicates that perturbations in the intratumoral microbiota can lead to abnormalities in fatty acid metabolic pathways. This, in turn, regulates stromal cell populations (such as fibroblasts), influencing patient prognosis and drug sensitivity (41). Additionally, the enrichment of bacteria related to bile acid metabolism (such as *Bilophila*) in CRC mucosa, along with its correlation with bile secretion gene expression, suggests a potential role for secondary bile acids in the carcinogenic process (27).

*F. nucleatum* also participates in the remodeling of amino acid metabolism. For instance, it promotes tumor cell proliferation by producing putrescine (42). It also metabolizes tryptophan to produce indole derivatives (such as 3-indolepropionic acid). These derivatives activate the aryl hydrocarbon receptor (AhR) signaling pathway, thereby promoting tumor stemness, epithelial-mesenchymal transition (EMT), and chemotherapy resistance (43–45). Conversely, colibactin-producing *E. coli* (*pks*+*E. coli*) can remodel lipid metabolism. This leads to a reduction in immunogenic lipids and the formation of a glycerophospholipid microenvironment within the tumor. This state of lipid overload not only hinders CD8+ T cell infiltration but also provides necessary energy for tumor cell survival, resulting in chemotherapy resistance (46). These metabolites serve not only as energy sources or synthetic raw materials but also as signaling molecules that regulate host cell transcriptional programs.

Regarding epigenetic regulation, gut and intratumoral microbes have been found to cause widespread changes in DNA methylation patterns in CRC tissues by influencing methyl donor metabolism. This is particularly evident in the abnormal methylation of promoter regions in tumor-related genes (47, 48). Although short-chain fatty acids (especially butyrate) are generally considered beneficial metabolites, their effects can be detrimental in specific spatial microenvironments within the tumor. Their inhibition of histone deacetylases (HDACs) may lead to epigenetic remodeling and exhaustion of CD8+ T cells, thereby producing an immunosuppressive effect (49). This “microbe-epigenetic” axis provides a new perspective for understanding how environmental factors induce changes in gene expression.

### 5.4 Regulation of the immune microenvironment

Intratumoral microbes are key drivers in reshaping the TME. *F. nucleatum* has been confirmed to interact with cancer-associated fibroblasts (CAFs). It induces CAFs to secrete pro-inflammatory cytokines (such as CXCL1 and IL-6), thereby promoting tumor cell invasion and metastasis (50). At the cellular immunity level, the presence of intratumoral microbes is closely related to the functional state of T cells. For instance, a high load of ETBF is associated with a decrease in FOXP3+ regulatory T cells (Tregs) and an increase in CD68+ macrophages (9). While seemingly contradictory, this likely reflects a complex immune escape mechanism. *Peptostreptococcus anaerobius* activates the integrin α2/β1- nuclear factor kappa B (NF-κB) signaling pathway in tumor cells to induce CXCL1 secretion. This recruits polymorphonuclear myeloid-derived suppressor cells (MDSCs). Simultaneously, the lytC_22 protein secreted by this bacterium acts directly on the Slamf4 receptor on MDSCs. This promotes their immunosuppressive activity, thereby weakening the anti-tumor response of T cells (51). On the other hand, specific microbial metabolites (such as the sphingolipid S1P) can upregulate PD-L1 (programmed death-ligand 1) expression in tumor cells. This leads to restricted expansion of effector T cells and an increase in exhausted T cells (52). Furthermore, ectopic colonization by oral pathogens (such as *Fusobacterium*) has been found to directly reduce CD8+ T cell infiltration by activating the TLR2/NF-κB pathway, thus weakening anti-tumor immune surveillance (53).

### 5.5 Selective shaping of intratumoral microbial colonization by genetic mutations

The host’s genetic background, particularly mutations in key driver genes, plays a selective role in shaping the colonization of intratumoral microbes. *KRAS* is a common mutated gene in CRC. Its mutation status is closely related to the composition of gut and intratumoral microbes. Studies have found that *KRAS* mutations inhibit the secretion of interferon-beta (IFN-β) by regulating specific microRNAs (such as miR3655) and their downstream targets (such as the SURF6/IRF7 axis). This reduces the host’s ability to clear ETBF, leading to increased ETBF colonization within the tumor (54). Conversely, the enrichment of ETBF further promotes tumor progression, creating a vicious cycle. Another study confirmed that the microbial community structure in tumor biopsies with *KRAS* mutations differs significantly from that in wild-type tumors. This heterogeneity is correlated with tumor progression (55). Additionally, single nucleotide polymorphisms (SNPs) in the host gene *KCNJ11* have been associated with *Fusobacterium* abundance. Carriers of specific alleles exhibit downregulated *KCNJ11* expression. This increases levels of Gal-GalNAc on the tumor cell surface, thereby promoting the adhesion and colonization of *F. nucleatum* via the Fap2 protein (56). These findings reveal a complex bidirectional interaction mechanism between host genetics and the intratumoral microecology.

## 6 Differential distribution of intratumoral microbiota across clinical-pathological features and molecular subtypes

### 6.1 Covariation patterns between consensus molecular subtypes and specific microbial communities

The molecular heterogeneity of CRC is closely coupled with the structure of its intratumoral microbial community. Studies indicate a significant covariation relationship between the widely used consensus molecular subtypes (CMS) classification system and specific microbial signatures. For instance, the CMS1 subtype (immune-activated) is typically associated with high microsatellite instability (MSI-H) and a high tumor mutation burden. In this subtype, dysregulated ferroptosis triggered by abundant immune cells is often observed, which may be linked to the colonization of specific microbes (57). The most significant association appears in the CMS4 subtype (mesenchymal, worst prognosis). Research has found that ETBF is significantly enriched in tumors of this subtype. Furthermore, experiments demonstrate that ETBF can induce tumor cells to exhibit CMS4-like gene expression profiles, thereby promoting epithelial-mesenchymal transition (EMT) and tumor growth (36). Additionally, distinct immune subtypes are also related to microbial communities. For example, in the IFN-γ-dominated C2 immune subtype, pathogenic genera such as *Selenomonas* and *Butyricimonas* are significantly enriched. This may explain the “immune paradox” observed in patients with this subtype, who exhibit poor survival rates despite high immune infiltration (58).

In specific clinical subgroups, the intratumoral microbiome also exhibits unique fingerprint characteristics. For example, in patients with colorectal cancer liver metastasis (CRLM), the intratumoral microbiome can be classified into three subtypes (IMCS1-3). These correspond to features related to glucose, protein, and lipid metabolism, respectively. Among them, the lipid metabolism-related subtype (IMCS3) is characterized by immune depletion and high invasiveness, carrying the worst prognosis (25). Furthermore, in appendiceal cancer, a unique pathological entity, the relationship between intratumoral *F. nucleatum* density and prognosis is contrary to that in CRC. High density is associated with better survival, suggesting organ-specific microbial mechanisms (59). Proteomic studies have also identified four novel CRC subtypes (C1-C4). These subtypes possess unique clinical prognoses and microbial features, revealing dynamic reorganization of host-microbe interactions at the protein level during the adenoma-to-carcinoma sequence (3).

### 6.2 Age-related characteristics: differences in intratumoral microecology between early-onset and late-onset CRC

With the rising incidence of early-onset colorectal cancer (EOCRC, onset age < 50 years), its unique etiological characteristics have attracted widespread attention. Differences in intratumoral microecology represent a crucial dimension. Compared with late-onset colorectal cancer (AOCRC), the tumor microbiota in EOCRC patients exhibits significantly different α-diversity and β-diversity (38). In EOCRC tumors, the relative abundances of *Akkermansia* and *Bacteroides* are higher. In contrast, *Fusobacterium* and *Bacillus* are more common in AOCRC (38). Although some studies suggest that differences in microbial composition between the two groups lack statistical consistency, the interaction patterns between microbes and the immune system in EOCRC show stronger and more extensive positive correlations. This suggests that immune microenvironment dysregulation is more prominent in EOCRC (47). Additionally, EOCRC patients typically exhibit accelerated epigenetic aging (DNA methylation age). This may also be related to specific microbial exposures and associated chronic inflammation (47). Another study focusing on young patients found that *Fusobacterium* acts as a key microbe in EOCRC. By co-localizing with cancer-associated fibroblasts (CAFs) and activating inflammatory pathways, it promotes tumorigenesis (53).

### 6.3 Gender and ethnic disparities in intratumoral microbiome distribution

Recent studies indicate an association between the intratumoral microbiome of CRC and host characteristics (such as gender and ethnicity), although current evidence remains incomplete. Regarding gender disparities, a large-cohort (n=859) droplet digital PCR (ddPCR) study revealed a significantly higher proportion of *F. nucleatum*-positive tumor tissues in female patients (60). However, because females are more prone to proximal colon cancer and *F. nucleatum* colonization exhibits an anatomical preference of increasing from the rectum to the cecum, tumor location serves as a strong covariate confounding this conclusion (61). As tumor location and molecular features may exert confounding effects on these results, further validation is required. Additionally, some small-sample studies suggest that the α-diversity in the female CRC group is higher than in males, with an observed enrichment of *Faecalibacterium*, *Megamonas*, and *Klebsiella* at the genus level in females; conversely, *Prevotella* and *Akkermansia* are more prominent in male CRC patients, and the phylum Fusobacteria is also higher in males (62). However, the cross-cohort reproducibility of these genus-level differences is poor, and these conclusions need to be confirmed in larger sample sizes.

Regarding ethnic disparities, a multinational study demonstrated significant differences in the composition of the CRC microbiome across different populations. For instance, African American CRC tissues are significantly enriched with *Leptotrichia*, European Americans are enriched with *Flexspiria* and *Streptococcus*, the Egyptian population is dominated by *Herbaspirillum* and *Staphylococcus*, while the Kenyan population exhibits an enrichment of *Akkermansia muciniphila* (63). Furthermore, a study on metastatic CRC combining RNA-seq suggested a higher abundance of actively transcribing bacteria, such as *Helicobacter cinaedi*, in the tumors of Black patients (64). These differences may be related to genetic backgrounds, dietary habits, and environmental factors. However, the sample sizes of existing studies are generally small, and the presence of batch effects and methodological discrepancies results in low reproducibility and reliability of the findings. Therefore, although current studies provide some clues, conclusions regarding gender and ethnic disparities still need to be validated in larger-scale prospective studies.

## 7 Clinical applications of the intratumoral microbiome in CRC

### 7.1 Application of the intratumoral and associated microbiome in early screening and diagnosis of CRC

Given the specific alterations of the microbiome in CRC, multimodal diagnostic strategies based on microbes are becoming a research hotspot. Fecal microbial detection holds the greatest potential for clinical translation due to its non-invasive nature. Multiple studies indicate that the abundance changes of key species, such as *F. nucleatum*, *Bacteroides fragilis*, and *Parvimonas micra*, can be combined with machine learning algorithms (e.g., random forest models). This approach allows for the construction of diagnostic models for CRC and adenoma with high sensitivity and specificity. Their performance often surpasses that of traditional fecal occult blood tests (FOBT) or carcinoembryonic antigen (CEA) detection (65–68). In particular, detection technologies for *F. nucleatum* are continuously innovating. Examples include droplet digital PCR (ddPCR) and CRISPR-Cas12a-based biosensors. These advancements have significantly improved detection limits for low-abundance samples, making the identification of early lesions possible (69, 70). Beyond fecal samples, the oral (saliva/plaque) microbiome also demonstrates unique diagnostic value. Due to the existence of the “oral-gut” axis, elevated levels of oral pathogens like *F. nucleatum* and *P. micra* in saliva have been confirmed to correlate with CRC risk. Furthermore, salivary microbial markers exhibit good diagnostic efficacy in distinguishing CRC patients from healthy individuals. They may even serve as effective supplements or alternatives to fecal testing (71, 72). This “distal” sampling approach offers new insights for non-invasive screening.

### 7.2 Clinical value of the intratumoral microbiome in the prognostic assessment of CRC

The intratumoral microbiome possesses not only diagnostic value but also serves as a crucial biomarker for CRC prognostic assessment. Extensive research consistently shows that high abundance of *F. nucleatum* is significantly associated with poor prognosis in CRC patients. This includes shorter overall survival (OS), shorter disease-free survival (DFS), and a higher risk of recurrence (73–75). This correlation is evident across different tumor stages. Particularly in stage III patients, intratumoral microbial characteristics are considered independent predictors of postoperative recurrence. Their predictive efficacy even outperforms traditional clinicopathological indicators (76). In metastatic CRC, the microbiome plays an equally critical role. Studies confirm that the persistent presence of *F. nucleatum* in metastatic lesions is significantly and positively correlated with lymph node and distant metastases. Additionally, the enrichment of ETBF corresponds directly to worse survival outcomes (53, 77). Moreover, specific microbial community subtypes (such as those characterized by glucose, protein, or lipid metabolism) have been identified as being associated with different prognoses in colorectal cancer liver metastasis (CRLM). This provides a new basis for risk stratification in CRLM (25). Beyond *F. nucleatum*, other microbes, such as specific species within *Bacteroides fragilis*, *Parvimonas micra*, and the *Alistipes* genus, have also been found to be associated with poor prognosis (78, 79).

### 7.3 Clinical value of the intratumoral microbiome in predicting therapeutic response in CRC

The characteristics of the intratumoral microbiota have emerged as important biomarkers for predicting responses to various colorectal cancer treatments. In the field of chemotherapy, direct microbial interference with drug metabolism is a significant cause of drug resistance. Studies have found that *F. nucleatum* is sensitive to the first-line chemotherapeutic agent 5-fluorouracil (5-FU). However, co-existing *Escherichia coli* possesses innate resistance to 5-FU. Furthermore, *E. coli* can detoxify 5-FU through metabolic modification, thereby protecting both *F. nucleatum* and tumor cells from drug-induced cytotoxicity (80). This synergistic protective mechanism between microbes explains the chemotherapy resistance observed in some patients. Additionally, models based on telomere scoring (TELscore) combined with microbiome features indicate that the high-score group (enriched with pathogens such as *Selenomonas*) exhibits reduced sensitivity to standard chemotherapy regimens (e.g., fluorouracil and oxaliplatin). Conversely, this group is more sensitive to MAPK pathway inhibitors. This suggests that microbial characteristics can guide the selection of chemotherapy regimens (81).

In the context of immune checkpoint inhibitor (ICI) therapy, the role of the intratumoral microbiome is particularly critical. Although *Helicobacter pylori* infection is primarily associated with gastric cancer, its positive status has been linked to the efficacy of immunotherapy in patients with dMMR/MSI-H (deficient mismatch repair/microsatellite instability-high) colorectal cancer. *H. pylori* infection remodels the structure of gut and intratumoral microbiota, such as the mutual exclusion observed between *Clostridium* and *Streptococcus* species. It enhances survival benefits from immunotherapy by augmenting amino acid metabolism and the abundance of specific bacterial groups (82). However, microbes can also act as barriers to immunotherapy. As previously mentioned, the microbial metabolite sphingosine-1-phosphate (S1P) can upregulate PD-L1. Interestingly, in the context of combination therapy with capecitabine, this might actually enhance the efficacy of anti-PD-1 antibodies by increasing the available targets. This complexity underscores the predictive value of microbial markers in specific combination therapies (52). Multiple studies have constructed microbiome-based scoring systems (such as the CMS score). These systems can effectively stratify patients, predict resistance to immunotherapy (indicated by high TIDE scores), and guide strategies such as combination with HDAC inhibitors to overcome drug resistance (49, 83).

For locally advanced rectal cancer (LARC), neoadjuvant chemoradiotherapy (nCRT) is the standard treatment, yet response rates vary significantly. Combined analysis of the intratumoral microbiome and immune gene expression profiles reveals that enrichment of genera such as *Fusobacterium*, *Coriobacteriaceae*, and *Sneathia* is closely associated with an incomplete response (Non-CR) to nCRT. In contrast, genera like *Lactobacillus* and *Streptococcus* are associated with a complete response (CR) (84). Further metagenomic analysis has identified key bacterial groups associated with radiation resistance, such as *Streptococcus equinus*, along with their metabolic functions (e.g., nitrate assimilation and histidine degradation). High-accuracy efficacy prediction models have subsequently been established (28). Interactions between microbes and cancer-associated fibroblasts (CAFs) have also been proven to mediate nCRT efficacy. Risk scores based on differential microbial signatures can independently predict treatment response (85). During chemoradiotherapy for anal squamous cell carcinoma, the enrichment of specific intratumoral genera (such as *Clostridia*) is closely associated with increased treatment toxicity (86). Additionally, tumor hypoxia induces specific microbial transcriptional responses, such as adaptive changes in *F. nucleatum*. This “hypoxia-microbe” interaction further reduces radiosensitivity and presages a poor prognosis (87).

## 8 Nanomedicine applications and therapeutic interventions targeting the intratumoral microbiome in CRC

**Figure 3:**
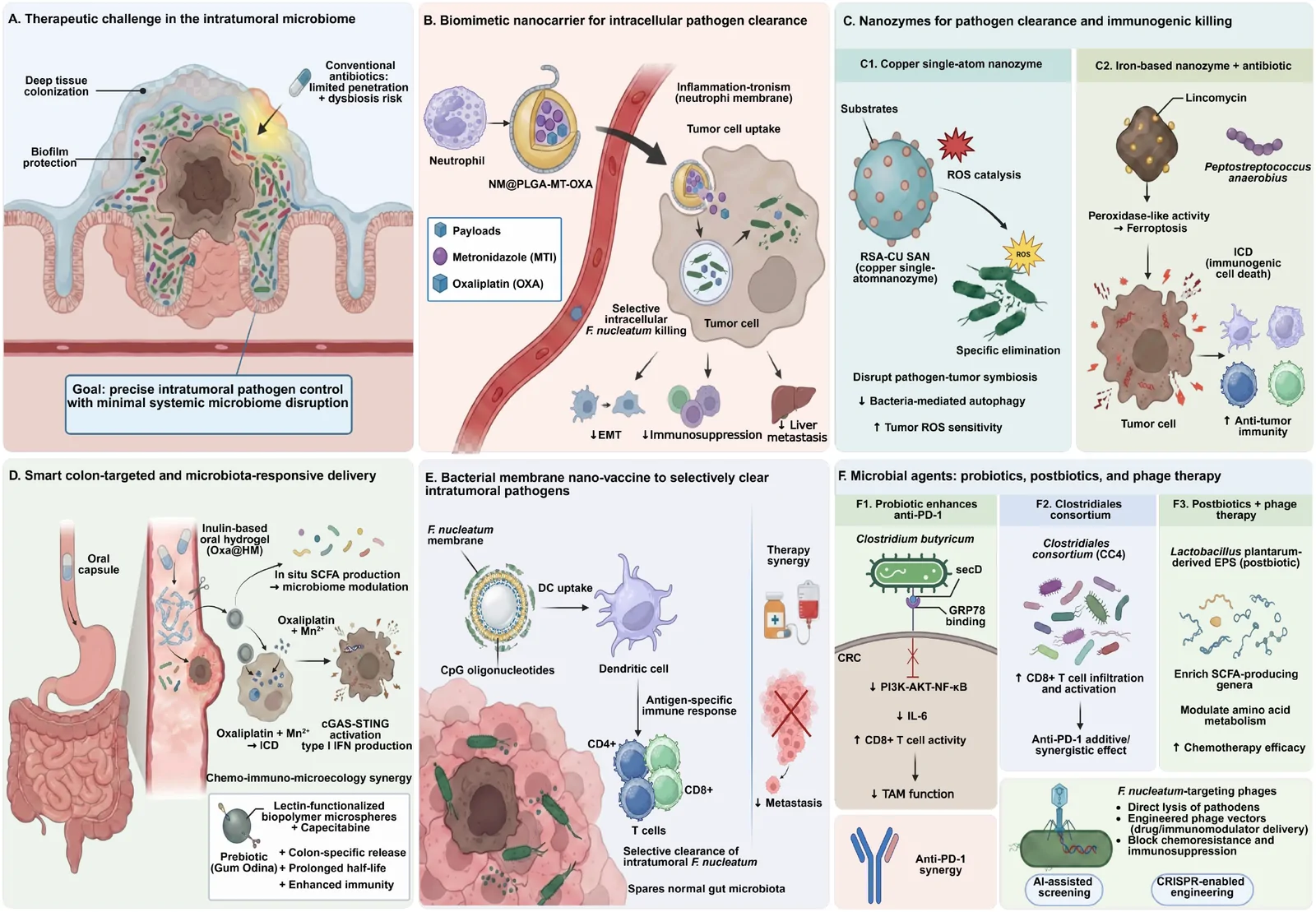
Therapeutic interventions targeting the intratumoral microbiome in CRC. **(A)** Therapeutic challenge in the intratumoral microbiome. Intratumoral microbes can colonize deep tumor tissues and are protected by biofilms, limiting antibiotic penetration and increasing the risk of systemic dysbiosis. The overarching goal is precise intratumoral pathogen control with minimal disruption of the host microbiome. **(B)** Biomimetic nanocarrier for intracellular pathogen clearance. Neutrophil membrane–coated poly (lactic-co-glycolic acid) nanoparticles co-loaded with metronidazole and oxaliplatin (NM@PLGA-MTI-OXA) exploit inflammation tropism to accumulate in tumors and be taken up by tumor cells, enabling selective killing of intracellular *F. nucleatum*. This strategy is depicted as reversing epithelial–mesenchymal transition (EMT) and reducing immunosuppression, thereby suppressing liver metastasis. **(C)** Nanozymes for pathogen clearance and immunogenic killing. (C1) A bovine serum albumin–supported copper single-atom nanozyme (BSA-Cu SAN) passively targets tumor sites and catalyzes reactive oxygen species (ROS) generation to specifically eliminate *F. nucleatum*, disrupt pathogen–tumor symbiosis, attenuate bacteria-mediated autophagy, and restore tumor sensitivity to ROS. (C2) A lincomycin-loaded iron-based nanozyme targets intratumoral *Peptostreptococcus anaerobius*; its peroxidase-like activity induces ferroptosis and triggers immunogenic cell death (ICD), promoting anti-tumor immunity. **(D)** Smart colon-targeted and microbiota-responsive delivery. An inulin-based oral hydrogel system (Oxa@HMI) is degraded by inulinase produced by colonic microbiota, releasing oxaliplatin-loaded hollow manganese oxide nanocarriers. This process supports *in situ* short-chain fatty acid (SCFA) production and microbiome modulation, while oxaliplatin and Mn²^+^ promote ICD and activate the cyclic GMP–AMP synthase–stimulator of interferon genes (cGAS–STING) pathway, achieving chemo–immuno–microecology synergy. A parallel strategy uses lectin-functionalized biopolymer microspheres loaded with capecitabine plus a prebiotic (Gum Odina) to enable colon-specific drug release, prolong half-life, and enhance anti-tumor immunity. **(E)** Bacterial membrane nano-vaccine to selectively clear intratumoral pathogens. A biomimetic nano-vaccine integrating *F. nucleatum* membranes with CpG oligodeoxynucleotides is taken up by dendritic cells (DCs), induces antigen-specific T-cell responses, and selectively clears intratumoral *F. nucleatum* while sparing normal gut microbiota, thereby improving chemotherapy efficacy and reducing metastasis. **(F)** Microbial agents: probiotics, postbiotics, and phage therapy. (F1) The probiotic *Clostridium butyricum* enhances anti-programmed cell death protein 1 (anti-PD-1) therapy via its surface protein secD binding to glucose-regulated protein 78 (GRP78), inhibiting phosphoinositide 3-kinase–protein kinase B–nuclear factor kappa-B (PI3K–AKT–NF-κB) signaling and decreasing interleukin-6 (IL-6), thereby increasing CD8+ T cell activity and weakening tumor-associated macrophage (TAM) function. (F2) A Clostridiales consortium (CC4) promotes intratumoral CD8+ T cell infiltration and activation and shows additive/synergistic effects with anti-PD-1. (F3) Lactobacillus plantarum–derived exopolysaccharides (EPS; postbiotic) enrich SCFA-producing genera and modulate amino-acid metabolism to enhance chemotherapy efficacy; engineered bacteriophages can directly lyse tumor-associated pathogens (including *F. nucleatum*) and serve as programmable vectors for drug/immunomodulator delivery, aided by artificial intelligence (AI)–assisted screening and clustered regularly interspaced short palindromic repeats (CRISPR)–enabled engineering. Created in BioRender. Steven, T. (2026) https://BioRender.com/eex0bgo.

Intratumoral microbes frequently reside in deep tumor regions and are shielded by biofilms, making them difficult to eradicate with conventional antibiotics while raising the risk of systemic dysbiosis. Accordingly, therapeutic strategies are shifting from broad antimicrobial suppression toward precision delivery and microecology modulation. This section summarizes nanotechnology-enabled platforms (engineered nanocarriers, nanozymes, and membrane-based nanovaccines) together with microbial-agent approaches (probiotics, postbiotics, and bacteriophages) for targeting the CRC intratumoral microbiome. An overview is provided in **Figure 3**.

### 8.1 Nanotechnology and engineered delivery systems: targeted pathogen clearance and synergistic drug efficacy

The introduction of nanotechnology and engineered delivery systems offers a breakthrough solution for precisely targeting the intratumoral microbiome. Strategies for clearing key intratumoral pathogens are becoming increasingly precise. Using biomimetic technology, researchers developed neutrophil membrane-coated PLGA nanoparticles co-loaded with metronidazole and oxaliplatin (NM@PLGA-MTI-OXA). These nanoparticles inherit the neutrophil’s tropism for inflammatory sites (tumors). They are efficiently taken up by tumor cells to precisely eliminate intracellular *F. nucleatum*. This reverses *F. nucleatum*-induced epithelial-mesenchymal transition (EMT) and immunosuppression, significantly inhibiting liver metastasis (23). Another strategy utilizes protein-supported copper single-atom nanozymes (BSA-Cu SAN). These nanozymes can passively target tumor sites. By catalyzing the production of reactive oxygen species (ROS), they specifically eliminate *F. nucleatum*, disrupting the “pathogen-tumor symbiote.” Simultaneously, they relieve bacteria-mediated autophagy activation, restoring tumor cell sensitivity to ROS (88). Additionally, a lincomycin-loaded iron-based nanozyme was designed to specifically target and eliminate intratumoral *Peptostreptococcus anaerobius*. It leverages peroxidase-like activity to induce ferroptosis in tumor cells. This triggers immunogenic cell death (ICD), thereby activating anti-tumor immunity (89).

Constructing smart responsive delivery systems can achieve synergistic enhancement of drugs and immunomodulators. An inulin-based oral hydrogel system (Oxa@HMI) was designed for colon-targeted delivery. This system utilizes inulinase produced by specific colonic flora to degrade the hydrogel backbone, releasing hollow manganese oxide nanocarriers loaded with oxaliplatin. This process generates short-chain fatty acids *in situ* to regulate the microecology. Furthermore, the released chemotherapeutic drugs and manganese ions induce immunogenic cell death and activate the cGAS-STING pathway. This achieves a synergistic effect combining chemotherapy, immunotherapy, and microecological regulation (90). Similarly, lectin-functionalized biopolymer microspheres loaded with capecitabine, combined with prebiotics (such as Gum Odina), can specifically release drugs in the colon. This regulates the flora, prolongs the drug half-life, and enhances anti-tumor immunity (91, 92).

Further research has explored nano-vaccine strategies based on bacterial membranes. A biomimetic nano-vaccine was constructed by integrating *F. nucleatum* bacterial membranes with adjuvants (such as CpG oligonucleotides). This vaccine is efficiently taken up by dendritic cells, inducing a strong specific immune response. It selectively clears intratumoral *F. nucleatum* without affecting normal intestinal flora, thereby significantly improving chemotherapy efficacy and reducing metastasis (93). This “bacteria-treating-bacteria” nanomedicine strategy solves the problem of antibiotic overuse. It also offers a new approach to overcoming bacteria-mediated drug resistance.

### 8.2 Innovative intervention strategies based on microbial agents: probiotics, postbiotics, and phage therapy

Probiotics and their engineered derivatives demonstrate significant therapeutic potential. *Clostridium butyricum* has been identified as a probiotic that enhances the efficacy of anti-PD-1 therapy. Its surface protein, secD, binds to the GRP78 receptor on the surface of CRC cells. This inhibits the PI3K-AKT-NF-κB pathway and reduces the secretion of the immunosuppressive cytokine IL-6. Consequently, it relieves inhibition of CD8+ T cells and weakens the function of tumor-associated macrophages (TAMs) (94). Additionally, a specific mixture of *Clostridiales* strains (CC4) has been confirmed to promote intratumoral infiltration and activation of CD8+ T cells. It exerts independent or synergistic anti-tumor effects with anti-PD-1 therapy in CRC models (95). To overcome the difficulty of colonizing oral probiotics, researchers developed exopolysaccharides (EPS) derived from *Lactobacillus plantarum*. This postbiotic regulates the gut microbiota and enriches short-chain fatty acid-producing genera. It also enhances chemotherapy efficacy by modulating amino acid metabolism (96).

Phage therapy is regaining attention as a highly specific antimicrobial method. Bacteriophages can directly lyse tumor-associated pathogens, including multidrug-resistant bacteria and *F. nucleatum*. They can also be engineered to serve as vectors for delivering drugs or immunomodulators. For example, specific phages targeting *F. nucleatum* can precisely clear the intratumoral pathogen load. This blocks bacteria-mediated chemotherapy resistance and immunosuppression. This “programmable” biological weapon, combined with AI-assisted screening and CRISPR technology, holds promise for achieving precise “surgical” modification of the tumor microecology (97).

## 9 Key technical limitations and challenges in current research

### 9.1 Detection difficulties in low-biomass samples and environmental contamination control

Compared to fecal samples, tumor tissue possesses a very low microbial biomass. This makes the “signal” easily masked by background “noise” from the environment. The so-called “Kitome” contamination—microbial DNA originating from extraction kits, PCR reagents, or the laboratory environment—represents one of the major technical challenges in this field (6, 13). Such contamination can lead to false-positive results. Consequently, distinguishing true tumor colonizers from environmental contaminants becomes extremely difficult. The lack of standardized pollution control protocols limits data comparability between different studies. This remains a key bottleneck hindering the translation of this field to clinical practice (98). To overcome this limitation, establishing strict experimental controls and replication systems is crucial. Nieciecki et al. demonstrated a workflow involving independent laboratory replication and the inclusion of multiple controls (such as blank and normal tissue controls). This approach effectively identified reproducible core microbial taxa, thereby eliminating false signals caused by environmental contamination (13).

### 9.2 Sampling heterogeneity and interference from gut microbiota

The unique anatomical structure of CRC makes it difficult to avoid contamination by luminal fecal flora when obtaining tumor tissues. Intestinal folds and villi can easily trap non-tumor-specific transient bacteria. This creates interference when defining the true “intratumoral” microbiome. Studies indicate that different sampling sites and tissue washing standards result in significant differences in the detected microbial composition (99). To address this issue, scholars have developed novel cell separation techniques, such as enzymatic digestion. These methods aim to remove non-specifically adherent bacteria, thereby more accurately enriching and detecting microbes closely associated with tumor cells. Results show that in enzymatically treated samples, the abundance of Proteobacteria increases significantly, while Firmicutes and Bacteroidetes decrease relative to untreated controls. This suggests that conventional bulk tissue sequencing may overestimate the contribution of feces-associated flora (99). Furthermore, tumors in different anatomical sites (such as the left versus right colon) possess distinct microbial baselines. This inherent heterogeneity necessitates detailed stratified analysis in study design (100).

### 9.3 Defining microbial colonization sources and causality

Although the presence of intratumoral microbes has been confirmed, their origins remain controversial. Current hypotheses include direct invasion following mucosal barrier damage, migration from adjacent tissues, hematogenous dissemination, and co-metastasis with tumor cells (6). In particular, how oral anaerobes such as *F. nucleatum* enrich in colorectal tumors requires more supporting evidence. A more central challenge lies in defining the causal relationship between microbes and tumors. Does a specific microbe drive tumorigenesis, or does the altered TME attract specific microbial colonization? We currently lack ideal models that fully simulate the complexity of the human TME. Additionally, interactions within microbial communities are complex. Consequently, current research often remains at the level of correlation analysis, making it difficult to establish definitive causal chains.

To bridge this gap and establish true causality, future methodologies must transition from observational studies to functional validations. Methodologically, researchers are increasingly relying on the inoculation of germ-free mice with defined synthetic microbial communities (SynComs) to track the temporal dynamics of tumorigenesis in a controlled environment. Furthermore, the application of Mendelian randomization to human genomic and metagenomic datasets offers a robust computational approach to infer causal directions, minimizing confounding biases. In terms of future directions, integrating high-resolution spatial transcriptomics with microbial single-cell RNA sequencing (scRNA-seq) will be vital. This will allow researchers to trace direct molecular crosstalk between specific microbes and host cells *in situ*. Additionally, the development of advanced organ-on-a-chip technologies that perfectly mimic the mucosal barrier and immune infiltration will provide the necessary physiological context to untangle these complex causal networks.

## 10 Conclusion and outlook

Research on the intratumoral microbiome in CRC is advancing from mere phenomenological description to a new stage of mechanistic elucidation and clinical translation. Intratumoral microbes are no longer viewed as meaningless background noise. Instead, they are recognized as indispensable functional components of the TME. Their heterogeneity in spatial distribution, along with their complex interactions with host genes and the immune system, profoundly influences the initiation, progression, and therapeutic outcomes of CRC. From the identification of core pathogens like *F. nucleatum* to the elucidation of mechanisms involving microbe-mediated metabolic reprogramming and immune escape, these findings provide abundant targets for the precision diagnosis and treatment of CRC.

In the future, resolving issues of contamination control in low-biomass samples and standardizing detection are prerequisites for clinical translation. Simultaneously, integrating multi-omics technologies, such as spatial transcriptomics and metabolomics, to comprehensively map microbe-host interactions will help reveal deeper molecular mechanisms. Developing microbiome-based precision intervention strategies, such as engineered probiotics and nanomedicine delivery systems, holds promise for bringing breakthrough progress to the comprehensive treatment of CRC. Ultimately, this will achieve the goal of improving patient prognosis. 

## References

[1] Bray F Laversanne M Sung H Ferlay J Siegel RL Soerjomataram I . Global cancer statistics 2022: GLOBOCAN estimates of incidence and mortality worldwide for 36 cancers in 185 countries. CA: Cancer J Clin. (2024) 74:229–63. doi: 10.3322/caac.21834 38572751

[2] Kennel KB Greten FR . The immune microenvironment of colorectal cancer. Nat Rev Cancer. (2025) 25:945–64. doi: 10.1038/s41568-025-00872-1 40983666

[3] Wu D Wang AJ Bu DC Sun YY Li CH Hong YM . The interplay between tissue-resident microbiome and host proteins by integrated multi-omics during progression of colorectal adenoma to carcinoma. Imeta. (2025) 4:e70090. doi: 10.1002/imt2.70090 41472855 PMC12747540

[4] Fan Y Gu X Yang H Chen Y Fang C Deng H . Intratumoral microbiome: a crucial regulating factor in development and progression of colorectal cancer. Mol BioMed. (2025) 6:138. doi: 10.1186/s43556-025-00376-2 41396345 PMC12705933

[5] Debelius JW Engstrand L Matussek A Brusselaers N Morton JT Stenmarker M . The local tumor microbiome is associated with survival in late-stage colorectal cancer patients. Microbiol Spectr. (2023) 11:e0506622. doi: 10.1128/spectrum.05066-22 37042765 PMC10269740

[6] Liao K Wen J Liu Z Zhang B Zhang X Fu Y . The role of intratumoral microbiome in the occurrence, proliferation, metastasis of colorectal cancer and its underlying therapeutic strategies. Ageing Res Rev. (2025) 111:102820. doi: 10.1016/j.arr.2025.102820 40639623

[7] Zong Z Zeng W Li Y Wang M Cao Y Cheng X . Intratumor microbiota and colorectal cancer: Comprehensive and lucid review. Chin J Cancer Res. (2024) 36:683–99. doi: 10.21147/j.issn.1000-9604.2024.06.07 39802896 PMC11724182

[8] Shakhpazyan NK Mikhaleva LM Sadykhov NK Midiber KY Buchaka AS Gioeva ZV . Tumor tissue microbiota in colorectal cancer: PCR profile of FFPE blocks in associations with metastatic status. Cells. (2025) 14:1508. doi: 10.3390/cells14191508 41090737 PMC12523663

[9] Saito R Shigematsu Y Amori M Amori G Takamatsu M Nishida K . Association of enterotoxigenic bacteroides fragilis with immune modulation in colorectal cancer liver metastasis. Cancers (Basel). (2025) 17:2733. doi: 10.3390/cancers17172733 40940828 PMC12427544

[10] Ou S Wang H Tao Y Luo K Ye J Ran S . Fusobacterium nucleatum and colorectal cancer: From phenomenon to mechanism. Front Cell Infect Microbiol. (2022) 12:1020583. doi: 10.3389/fcimb.2022.1020583 36523635 PMC9745098

[11] Wang Q Ding H . Synergistic interactions between gut and intratumoral microbiota: A new perspective on the oncogenesis and treatment of colorectal cancer. Dig Dis Sci. (2025). doi: 10.1007/s10620-025-09490-1 41137871

[12] Cao Y Wang Z Yan Y Ji L He J Xuan B . Enterotoxigenic Bacteroides fragilis Promotes Intestinal Inflammation and Malignancy by Inhibiting Exosome-Packaged miR-149-3p. Gastroenterology. (2021) 161:1552–1566.e1512. doi: 10.1053/j.gastro.2021.08.003 34371001

[13] Nieciecki VF Blum FC Johnson RC Testerman TL McAvoy TJ King MC . Cross-laboratory replication of pseudomyxoma peritonei tumor microbiome reveals reproducible microbial signatures. mSphere. (2025) 10:e0065224. doi: 10.1128/msphere.00652-24 39976448 PMC11934312

[14] Chauca-Bajana L Ordonez Balladares A Lorenzo-Pouso AI Caicedo-Quiroz R Erazo Vaca RX Dau Villafuerte RF . Periodontitis and oral pathogens in colorectal cancer: A systematic review, meta-analysis, and trial sequential analysis. Dent J (Basel). (2025) 13:595. doi: 10.3390/dj13120595 41440353 PMC12731773

[15] Zhu J Jiang Z Yu F Gao L Wang X Wang Q . Integrated oral-gut microbiota therapy: a novel perspective on preventing bacterial translocation for systemic disease management. Front Cell Infect Microbiol. (2025) 15:1641816. doi: 10.3389/fcimb.2025.1641816 40792109 PMC12336206

[16] Zhang X Wu M Shi H Kim S Lu S Wang P . Amplification-free electrochemiluminescent biosensor for ultrasensitive detection of fusobacterium nucleatum using tetrahedral DNA-based CRISPR/cas12a. Cyborg Bionic Syst. (2025) 6:0266. doi: 10.34133/cbsystems.0266 40313468 PMC12044220

[17] Zhu X Xu P Zhu R Gao W Yin W Lan P . Multi-kingdom microbial signatures in excess body weight colorectal cancer based on global metagenomic analysis. Commun Biol. (2024) 7:24. doi: 10.1038/s42003-023-05714-0 38182885 PMC10770074

[18] Jin M Shang F Wu J Fan Q Chen C Fan J . Tumor-Associated microbiota in proximal and distal colorectal cancer and their relationships with clinical outcomes. Front Microbiol. (2021) 12:727937. doi: 10.3389/fmicb.2021.727937 34650531 PMC8506159

[19] Phipps O Quraishi MN Dickson EA Steed H Kumar A Acheson AG . Differences in the on- and off-tumor microbiota between right- and left-sided colorectal cancer. Microorganisms. (2021) 9:1108. doi: 10.3390/microorganisms9051108 34065545 PMC8160982

[20] Garcia Menendez G Sichel L Lopez MDC Hernandez Y Arteaga E Rodriguez M . From colon wall to tumor niche: Unraveling the microbiome’s role in colorectal cancer progression. PloS One. (2024) 19:e0311233. doi: 10.1371/journal.pone.0311233 39436937 PMC11495602

[21] An HJ Partha MA Lee H Lau BT Pavlichin DS Almeda A . Tumor-associated microbiome features of metastatic colorectal cancer and clinical implications. Front Oncol. (2023) 13:1310054. doi: 10.3389/fonc.2023.1310054 38304032 PMC10833227

[22] Kneis B Wirtz S Weber K Denz A Gittler M Geppert C . Colon cancer microbiome landscaping: differences in right- and left-Sided colon cancer and a tumor microbiome-Ileal microbiome association. Int J Mol Sci. (2023) 24:3265. doi: 10.3390/ijms24043265 36834671 PMC9963782

[23] Niu Y Zhao X Li Y Ma X Yang W Ma J . Neutrophil-mimicking nanomedicine eliminates tumor intracellular bacteria and enhances chemotherapy on liver metastasis of colorectal cancer. Advanced Sci (Weinheim Baden-Wurttemberg Germany). (2025) 12:e04188. doi: 10.1002/advs.202504188 40433907 PMC12376532

[24] Hilmi M Kamal M Vacher S Dupain C Ibadioune S Halladjian M . Intratumoral microbiome is driven by metastatic site and associated with immune histopathological parameters: An ancillary study of the SHIVA clinical trial. Eur J Cancer. (2023) 183:152–61. doi: 10.1016/j.ejca.2023.01.024 36868056

[25] Yan L Wei X Zhong F Fu L Ru H Mo X . Intratumoral microbial community profiling identifies clinicomolecular and prognostic subtypes of colorectal cancer liver metastasis. NPJ Precis Oncol. (2025) 9:284. doi: 10.1038/s41698-025-01075-5 40813885 PMC12354738

[26] Shi Y Liu L Wu J Gao M Sheng K Hou W . Whole-genome sequencing and analysis of a novel strain Streptococcus oralis CRC211 from colorectal tumor. Microbiome Res Rep. (2025) 4:33. doi: 10.20517/mrr.2025.41 41133102 PMC12540049

[27] Qin Y Wang Q Lin Q Liu F Pan X Wei C . Multi-omics analysis reveals associations between gut microbiota and host transcriptome in colon cancer patients. mSystems. (2025) 10:e0080524. doi: 10.1128/msystems.00805-24 40013792 PMC11915798

[28] Huang X Chen C Xie W Zhou C Tian X Zhang Z . Metagenomic analysis of intratumoral microbiome linking to response to neoadjuvant chemoradiotherapy in rectal cancer. Int J Radiat oncology biology Phys. (2023) 117:1255–69. doi: 10.1016/j.ijrobp.2023.06.2515 37433373

[29] Yu C Zhou Z Liu B Yao D Huang Y Wang P . Investigation of trends in gut microbiome associated with colorectal cancer using machine learning. Front Oncol. (2023) 13:1077922. doi: 10.3389/fonc.2023.1077922 36937384 PMC10015000

[30] Galeano Nino JL Wu H LaCourse KD Kempchinsky AG Baryiames A Barber B . Effect of the intratumoral microbiota on spatial and cellular heterogeneity in cancer. Nature. (2022) 611:810–7. doi: 10.1038/s41586-022-05435-0 36385528 PMC9684076

[31] Robinson W Stone JK Schischlik F Gasmi B Kelly MC Seibert C . Identification of intracellular bacteria from multiple single-cell RNA-seq platforms using CSI-Microbes. Sci Adv. (2024) 10:eadj7402. doi: 10.1126/sciadv.adj7402 38959321 PMC11221508

[32] Richter KM Wrage M Krekeler C De Oliveira T Conradi LC Menck K . Model systems to study tumor-microbiome interactions in early-onset colorectal cancer. EMBO Mol Med. (2025) 17:395–413. doi: 10.1038/s44321-025-00198-3 39948421 PMC11903813

[33] Nalluri H Subramanian S Staley C . Intestinal organoids: a model to study the role of microbiota in the colonic tumor microenvironment. Future Microbiol. (2020) 15:1583–94. doi: 10.2217/fmb-2019-0345 33215543

[34] Wang LJ Mo YK Cheng Y . The role of intratumoral microbiota in the occurrence and progression of tumors and its implications for guiding tumor treatment. Acta Microbiol Immunol Hung. (2025). doi: 10.1556/030.2025.02747 41342899

[35] Kitano T Okumura S Matsumoto T Tsunoda S Nishigori T Obama K . The Involvement of Oral Pathogenic Bacteria, Fusobacterium nucleatum Subspecies animalis in the Pathogenesis of Human Esophageal Adenocarcinoma. Gastro Hep Adv. (2025) 4:100660. doi: 10.1016/j.gastha.2025.100660 40486267 PMC12144443

[36] Chang SY Park J Park SJ Park JJ Cheon JH Kim DK . Bacteroides fragilis promotes mesenchymal subtype in colorectal cancer. Cancers (Basel). (2025) 17:3822. doi: 10.3390/cancers17233822 41375022 PMC12691386

[37] El Tekle G Andreeva N Garrett WS . The role of the microbiome in the etiopathogenesis of colon cancer. Annu Rev Physiol. (2024) 86:453–78. doi: 10.1146/annurev-physiol-042022-025619 38345904

[38] Barot SV Sangwan N Nair KG Schmit SL Xiang S Kamath S . Distinct intratumoral microbiome of young-onset and average-onset colorectal cancer. EBioMedicine. (2024) 100:104980. doi: 10.1016/j.ebiom.2024.104980 38306898 PMC10850116

[39] Shen W Li Z Wang L Liu Q Zhang R Yao Y . Tumor-resident Malassezia can promote hepatocellular carcinoma development by downregulating bile acid synthesis and modulating tumor microenvironment. Sci Rep. (2025) 15:15020. doi: 10.1038/s41598-025-99973-y 40301518 PMC12041395

[40] Geng L Fan Z Chen R Cho KC Liu Y Cheng Y . The Nalpha-acetyl-L-lysine/Loxl2/H(2)O(2) promotes intestinal tumor growth in Drosophila and cell proliferation in human colorectal cancer. Cell Rep. (2025) 44:116126. doi: 10.1016/j.celrep.2025.116126 40811058

[41] Liu G Liu K Ji L Li Y . Intratumoral microbiota, fatty acid metabolism, and tumor microenvironment constitute an unresolved trinity in colon adenocarcinoma. Sci Rep. (2025) 15:2568. doi: 10.1038/s41598-025-87194-2 39833403 PMC11747563

[42] Xu S CB G Phan A Wu C . The gene encoding ornithine decarboxylase for putrescine biosynthesis is essential for the viability of Fusobacterium nucleatum. J bacteriology. (2025) 208:e0038725. doi: 10.1101/2025.09.02.673652 41347511 PMC12826042

[43] Song Q Jin Z Zhang H Hong K Zhu B Yin H . Fusobacterium nucleatum-derived 3-indolepropionic acid promotes colorectal cancer progression via aryl hydrocarbon receptor activation in macrophages. Chemico-biological Interact. (2025) 414:111495. doi: 10.1016/j.cbi.2025.111495 40174685

[44] Zalila-Kolsi I Dhieb D Osman HA Mekideche H . The gut microbiota and colorectal cancer: understanding the link and exploring therapeutic interventions. Biol (Basel). (2025) 14:251. doi: 10.3390/biology14030251 40136508 PMC11939563

[45] Tesolato S Vicente-Valor J Paz-Cabezas M Gomez-Garre D Sanchez-Gonzalez S Ortega-Hernandez A . Gut microbiota signatures with potential clinical usefulness in colorectal and non-Small cell lung cancers. Biomedicines. (2024) 12:703. doi: 10.3390/biomedicines12030703 38540316 PMC10967942

[46] de Oliveira Alves N Dalmasso G Nikitina D Vaysse A Ruez R Ledoux L . The colibactin-producing Escherichia coli alters the tumor microenvironment to immunosuppressive lipid overload facilitating colorectal cancer progression and chemoresistance. Gut Microbes. (2024) 16:2320291. doi: 10.1080/19490976.2024.2320291 38417029 PMC10903627

[47] Jin N Hoyd R Yilmaz AS Zhu J Liu Y Jagjit Singh MS . Epigenetic modulation, intratumoral microbiome, and immunity in early-Onset colorectal cancer. Cancer Res Commun. (2025) 5:1985–97. doi: 10.1158/2767-9764.CRC-25-0177 41134679 PMC12606411

[48] Liu Z Zhang Q Zhang H Yi Z Ma H Wang X . Colorectal cancer microbiome programs DNA methylation of host cells by affecting methyl donor metabolism. Genome Med. (2024) 16:77. doi: 10.1186/s13073-024-01344-1 38840170 PMC11151592

[49] Chen X Zhang Y Zhang G Wang D Dou L Wang Y . Spatial microbiome-metabolic crosstalk drives CD8(+) T-cell exhaustion through the butyrate-HDAC axis in colorectal cancer. Front Microbiol. (2025) 16:1704491. doi: 10.3389/fmicb.2025.1704491 41438381 PMC12719255

[50] Karta J Meyers M Rodriguez F Koncina E Gilson C Klein E . Fusobacterium nucleatum interacts with cancer-associated fibroblasts to promote colorectal cancer. EMBO J. (2025) 44:5375–93. doi: 10.1038/s44318-025-00542-w 40846900 PMC12488894

[51] Liu Y Wong CC Ding Y Gao M Wen J Lau HC . Peptostreptococcus anaerobius mediates anti-PD1 therapy resistance and exacerbates colorectal cancer via myeloid-derived suppressor cells in mice. Nat Microbiol. (2024) 9:1467–82. doi: 10.1038/s41564-024-01695-w 38750176 PMC11153135

[52] Dai CS Qi TT Shang HL Xie RH Liu H Liu ZM . Intratumoral microbiota-derived S1P sensitizes the combination therapy of capecitabine and PD-1 inhibitors. iScience. (2025) 28:114202. doi: 10.1016/j.isci.2025.114202 41446733 PMC12723371

[53] Xu Z Lv Z Chen F Zhang Y Xu Z Huo J . Dysbiosis of human tumor microbiome and aberrant residence of Actinomyces in tumor-associated fibroblasts in young-onset colorectal cancer. Front Immunol. (2022) 13:1008975. doi: 10.3389/fimmu.2022.1008975 36119074 PMC9481283

[54] Chen Y Liu S Tan S Zheng Y Chen Y Yang C . KRAS mutations promote the intratumoral colonization of enterotoxigenic bacteroides fragilis in colorectal cancer through the regulation of the miRNA3655/SURF6/IRF7/IFNbeta axis. Gut Microbes. (2024) 16:2423043. doi: 10.1080/19490976.2024.2423043 39523457 PMC11556274

[55] Liu W Zhang X Xu H Li S Lau HC Chen Q . Microbial community heterogeneity within colorectal neoplasia and its correlation with colorectal carcinogenesis. Gastroenterology. (2021) 160:2395–408. doi: 10.1053/j.gastro.2021.02.020 33581124

[56] Chen Q Wang Y Wu D Zhang H Xia Q Qu D . Integrated microbiome-metabolome profiling unveils a predictive signature for early recurrence in hepatocellular carcinoma. Front Microbiol. (2025) 16. doi: 10.3389/fmicb.2025.1653249 40969429 PMC12442831

[57] Li X Wu D Li Q Gu J Gao W Zhu X . Host-microbiota interactions contributing to the heterogeneous tumor microenvironment in colorectal cancer. Physiol Genomics. (2024) 56:221–34. doi: 10.1152/physiolgenomics.00103.2023 38073489

[58] Jemimah S Majdalawieh AF Hamad M Mahasneh AA . Differential analysis of microbial profiles in colorectal cancer reveals modulations corresponding to immune subtypes. Comput Biol Med. (2026) 200:111346. doi: 10.1016/j.compbiomed.2025.111346 41343929

[59] Sherry C Dadgar N Park H Knotts C Grayhack E Blodgett R . Fusobacterium nucleatum is associated with tumor characteristics, immune microenvironment, and survival in appendiceal cancer. Microorganisms. (2025) 13:1644. doi: 10.3390/microorganisms13071644 40732152 PMC12299451

[60] Phipps AI Hill CM Lin G Malen RC Reedy AM Kahsai O . Fusobacterium nucleatum enrichment in colorectal tumor tissue: associations with tumor characteristics and survival outcomes. Gastro hep Adv. (2025) 4:100644. doi: 10.1016/j.gastha.2025.100644 40487272 PMC12138897

[61] Mima K Cao Y Chan AT Qian ZR Nowak JA Masugi Y . Fusobacterium nucleatum in colorectal carcinoma tissue according to tumor location. Clin Trans Gastroenterol. (2016) 7:e200. doi: 10.1038/ctg.2016.53 27811909 PMC5543402

[62] Costa CPD Vieira P Mendes-Rocha M Pereira-Marques J Ferreira RM Figueiredo C . The tissue-associated microbiota in colorectal cancer: A systematic review. Cancers (Basel). (2022) 14:3385. doi: 10.3390/cancers14143385 35884445 PMC9317273

[63] Elkholy A Avuthu N Abdalla M Behring M Bajpai P Kim H-G . Microbiome diversity in African American, European American, and Egyptian colorectal cancer patients. Heliyon. (2023) 9:e18035. doi: 10.1016/j.heliyon.2023.e18035 37483698 PMC10362239

[64] Feng L Wang R Zhao Q Wang J Luo G Xu C . Racial disparities in metastatic colorectal cancer outcomes revealed by tumor microbiome and transcriptome analysis with bevacizumab treatment. Front Pharmacol. (2023) 14:1320028. doi: 10.3389/fphar.2023.1320028 38357363 PMC10864621

[65] Bai B Ma J Xu W Chen X Chen X Lv C . Gut microbiota and colorectal cancer: mechanistic insights, diagnostic advances, and microbiome-based therapeutic strategies. Front Microbiol. (2025) 16:1699893. doi: 10.3389/fmicb.2025.1699893 41356485 PMC12675188

[66] Schulz C Vilchez-Vargas R Öcal E Koch N Puhr-Westerheide D Burnell LF . Profiling of the tumor-associated microbiome in patients with hepatocellular carcinoma. Gut Pathog. (2025) 17:53. doi: 10.1186/s13099-025-00727-y 40635009 PMC12243435

[67] Goto Y Iwata S Miyahara M Miyako E . Discovery of intratumoral oncolytic bacteria toward targeted anticancer theranostics. Adv Sci (Weinh). (2023) 10:e2301679. doi: 10.1002/advs.202301679 37150857 PMC10369285

[68] Novielli P Baldi S Romano D Magarelli M Diacono D Di Bitonto P . Personalized colorectal cancer risk assessment through explainable AI and Gut microbiome profiling. Gut Microbes. (2025) 17:2543124. doi: 10.1080/19490976.2025.2543124 40760681 PMC12326576

[69] Seo Y Kim KA Lee S Lim YH Seo Y Kim TK . A Duplex qPCR Assay Targeting the fadA Gene Enables Robust Detection of Fusobacterium in Clinical Samples. Int J Mol Sci. (2025) 26:11319. doi: 10.3390/ijms262311319 41373480 PMC12691792

[70] Wang X Feng S Chen H Zhou B Fan T Qin Y . Development of an aptamer/CRISPR-cas12a-based dual-modal biosensor for fusobacterium nucleatum detection in non-invasive colorectal cancer screening. Analytical Chem. (2025) 97:23360–9. doi: 10.1021/acs.analchem.5c04132 41081763

[71] Krokosz S Obrycka M Zalewska A . Can salivary biomarkers serve as diagnostic and prognostic tools for early detection in patients with colorectal cancer? A systematic review. Curr Issues Mol Biol. (2025) 47:647. doi: 10.3390/cimb47080647 40864801 PMC12384429

[72] Nawab S Bao Q Ji LH Luo Q Fu X Fan S . The pathogenicity of fusobacterium nucleatum modulated by dietary fibers-A possible missing link between the dietary composition and the risk of colorectal cancer. Microorganisms. (2023) 11:2004. doi: 10.3390/microorganisms11082004 37630564 PMC10458976

[73] Wang Q Chen C Zhao H Jiao Y Chen H Wang P . Magnetotactic bacteria-mediated integrated magnetic targeted hyperthermia for *in-situ* deep-seated tumor. Colloids Surfaces B: Biointerfaces. (2025) 252:114658. doi: 10.1016/j.colsurfb.2025.114658 40168695

[74] Kulmambetova G Kurentay B Gusmaulemova A Utupov T Auganova D Tarlykov P . Association of Fusobacterium nucleatum infection with colorectal cancer in Kazakhstani patients. Front Oncol. (2024) 14:1473575. doi: 10.3389/fonc.2024.1473575 39726700 PMC11669545

[75] Mouradov D Greenfield P Li S In EJ Storey C Sakthianandeswaren A . Oncomicrobial community profiling identifies clinicomolecular and prognostic subtypes of colorectal cancer. Gastroenterology. (2023) 165:104–20. doi: 10.1053/j.gastro.2023.03.205 36933623

[76] Li Z Liu Y Guo P Wei Y . Construction and validation of a novel angiogenesis pattern to predict prognosis and immunotherapy efficacy in colorectal cancer. Aging (Albany NY). (2023) 15:12413–50. doi: 10.18632/aging.205189 37938164 PMC10683615

[77] Zhang C Hu A Li J Zhang F Zhong P Li Y . Combined non-invasive prediction and new biomarkers of oral and fecal microbiota in patients with gastric and colorectal cancer. Front Cell Infect Microbiol. (2022) 12:830684. doi: 10.3389/fcimb.2022.830684 35663463 PMC9161364

[78] Hasan R Shaikh MTM Rawat S Singh V Tamang R Choudhury S . Intratumoral microbiome signatures in a North Central Indian colorectal cancer cohort: identification of novel prognostic biomarkers and functional pathways. Sci Rep. (2026) 16:1815. doi: 10.1038/s41598-025-31383-6 41526416 PMC12804881

[79] Qu R Zhang Y Ma Y Zhou X Sun L Jiang C . Role of the gut microbiota and its metabolites in tumorigenesis or development of colorectal cancer. Adv Sci (Weinh). (2023) 10:e2205563. doi: 10.1002/advs.202205563 37263983 PMC10427379

[80] LaCourse KD Zepeda-Rivera M Kempchinsky AG Baryiames A Minot SS Johnston CD . The cancer chemotherapeutic 5-fluorouracil is a potent Fusobacterium nucleatum inhibitor and its activity is modified by intratumoral microbiota. Cell Rep. (2022) 41:111625. doi: 10.1016/j.celrep.2022.111625 36384132 PMC10790632

[81] Zhang Y Fan J Zhao J Zhu H Xia Y Xu H . A telomere-associated molecular landscape reveals immunological, microbial, and therapeutic heterogeneity in colorectal cancer. Front Mol Biosci. (2025) 12:1615533. doi: 10.3389/fmolb.2025.1615533 40492114 PMC12146184

[82] Jia K Chen Y Dai D Xie Y Peng H Cao Y . Impact of Helicobacter pylori infection on gut and intratumoral microbiome and its association with immunotherapy response in gastrointestinal cancer. BMC Med. (2026) 24:79. doi: 10.1186/s12916-025-04575-0 41526899 PMC12888622

[83] Omar TM Al-Hussainy AF Jyothi SR Priyadarshini Nayak P Bethanney Janney J Singh G . Invisible influencers: the tumor microbiome’s impact on immunotherapy in colorectal cancer (CRC). Expert Rev Anticancer Ther. (2025) 26:161–178. doi: 10.1080/14737140.2025.2579656 41123566

[84] Roesel R Strati F Basso C Epistolio S Spina P Djordjevic J . Combined tumor-associated microbiome and immune gene expression profiling predict response to neoadjuvant chemo-radiotherapy in locally advanced rectal cancer. Oncoimmunology. (2025) 14:2465015. doi: 10.1080/2162402X.2025.2465015 39992705 PMC11853554

[85] Sun L Qu J Ke X Zhang Y Xu H Lv N . Interaction between intratumoral microbiota and tumor mediates the response of neoadjuvant therapy for rectal cancer. Front Microbiol. (2023) 14:1229888. doi: 10.3389/fmicb.2023.1229888 37901832 PMC10602640

[86] Lin D El Alam MB Jaoude JA Kouzy R Phan JL Elnaggar JH . Microbiome dynamics during chemoradiation therapy for anal cancer. Int J Radiat oncology biology Phys. (2022) 113:974–84. doi: 10.1016/j.ijrobp.2022.04.037 35513187

[87] Benej M Hoyd R Kreamer M Wheeler CE Grencewicz DJ Choueiry F . The tumor microbiome reacts to hypoxia and can influence response to radiation treatment in colorectal cancer. Cancer Res Commun. (2024) 4:1690–701. doi: 10.1158/2767-9764.CRC-23-0367 38904265 PMC11234499

[88] Wang X Chen Q Zhu Y Wang K Chang Y Wu X . Destroying pathogen-tumor symbionts synergizing with catalytic therapy of colorectal cancer by biomimetic protein-supported single-atom nanozyme. Signal Transduct Target Ther. (2023) 8:277. doi: 10.1038/s41392-023-01491-8 37474504 PMC10359331

[89] Cao Y Wang J Wang H Sun X Fang F Li S . Targeting peptostreptococcus anaerobius with an iron-based nanozyme reverses ferroptosis resistance and enhances antitumor immunity in colorectal cancer. Adv Sci (Weinh). (2026), e16272. doi: 10.1002/advs.202516272 41524179 PMC13292267

[90] Li L He S Liao B Wang M Lin H Hu B . Orally administrated hydrogel harnessing intratumoral microbiome and microbiota-Related immune responses for potentiated colorectal cancer treatment. Res (Wash D C). (2024) 7:0364. doi: 10.34133/research.0364 38721274 PMC11077293

[91] Hazra A Tudu M Mohanta A Samanta A . Gum odina prebiotic induced gut modulation for the treatment of colon cancer on Swiss albino mice: By using capecitabine loaded biopolymeric microsphere. Int J Biol Macromol. (2024) 267:131410. doi: 10.1016/j.ijbiomac.2024.131410 38582484

[92] Lang T Zhu R Zhu X Yan W Li Y Zhai Y . Combining gut microbiota modulation and chemotherapy by capecitabine-loaded prebiotic nanoparticle improves colorectal cancer therapy. Nat Commun. (2023) 14:4746. doi: 10.1038/s41467-023-40439-y 37550297 PMC10406894

[93] Chen L Kang Z Shen J Zhao R Miao Y Zhang L . An emerging antibacterial nanovaccine for enhanced chemotherapy by selectively eliminating tumor-colonizing bacteria. Sci Bull (Beijing). (2024) 69:2565–79. doi: 10.1016/j.scib.2024.06.016 38918142

[94] Xie M Yuan K Zhang Y Zhang Y Zhang R Gao J . Tumor-resident probiotic Clostridium butyricum improves aPD-1 efficacy in colorectal cancer models by inhibiting IL-6-mediated immunosuppression. Cancer Cell. (2025) 43:1885–1901 e1810. doi: 10.1016/j.ccell.2025.07.012 40780216

[95] Montalban-Arques A Katkeviciute E Busenhart P Bircher A Wirbel J . Commensal Clostridiales strains mediate effective anti-cancer immune response against solid tumors. Cell host & microbe. (2021) 29:1573–1588.e1577. doi: 10.1016/j.chom.2021.08.001 34453895

[96] Chen M Bie L . Intratumoral microbiota for hepatocellular carcinoma: from preclinical mechanisms to clinical cancer treatment. Cancer Cell Int. (2025) 25(1):52. doi: 10.1186/s12935-025-03745-7 40247312 PMC12007317

[97] Hsu CY Polatova D Hamad RH Patel PN Akram M Singh G . Phage therapy in cancer treatment: Mechanisms, emerging innovations, and translational progress. Crit Rev Oncol Hematol. (2025) 218:105085. doi: 10.1016/j.critrevonc.2025.105085 41397585

[98] Li C Cai C Wang C Chen X Zhang B Huang Z . Gut microbiota-mediated gut-liver axis: a breakthrough point for understanding and treating liver cancer. Clin Mol Hepatol. (2025) 31:350–81. doi: 10.3350/cmh.2024.0857 39659059 PMC12016628

[99] Zuo Y Lu Y Pang J Jin S Zhang X Zhao E . Detection and comparison of tumor cell-associated microbiota from different compartments of colorectal cancer. Front Oncol. (2024) 14:1374769. doi: 10.3389/fonc.2024.1374769 38835371 PMC11148212

[100] Chen Y Liao X Li Y Cao H Zhang F Fei B . Effects of prebiotic supplement on gut microbiota, drug bioavailability, and adverse effects in patients with colorectal cancer at different primary tumor locations receiving chemotherapy: study protocol for a randomized clinical trial. Trials. (2023) 24:268. doi: 10.1186/s13063-023-07137-y 37046334 PMC10091326

[101] Purcell RV Visnovska M Biggs PJ Schmeier S Frizelle FA . Distinct gut microbiome patterns associate with consensus molecular subtypes of colorectal cancer. Sci Rep. (2017) 7:11590. doi: 10.1038/s41598-017-11237-6 28912574 PMC5599497

[102] Byrd DA Fan W Greathouse KL Wu MC Xie H Wang X . The intratumor microbiome is associated with microsatellite instability. J Natl Cancer Institute. (2023) 115:989–93. doi: 10.1093/jnci/djad083 37192013 PMC10407713

[103] Kushwaha M Dalal N Chaudhary S Ahmed A Makharia GK Singh AK . Colorectal cancer biofilm composition reveals distinct bacterial species signature. Appl Microbiol Biotechnol. (2025) 109:159. doi: 10.1007/s00253-025-13537-8 40603621 PMC12222442

[104] Parajuli B Midya V Kiddle R De Jager N Eggers S Spakowicz D . Primary tumor microbiomes predict distant metastasis of colorectal cancer. NPJ Precis Oncol. (2025) 9:405. doi: 10.1038/s41698-025-01188-x 41286005 PMC12748615

[105] Xu Y Zhao J Ma Y Liu J Cui Y Yuan Y . The microbiome types of colorectal tissue are potentially associated with the prognosis of patients with colorectal cancer. Front Microbiol. (2023) 14:1100873. doi: 10.3389/fmicb.2023.1100873 37025624 PMC10072283

[106] González A Fullaondo A Navarro D Rodríguez J Tirnauca C Odriozola A . New insights into mucosa-associated microbiota in paired tumor and non-tumor adjacent mucosal tissues in colorectal cancer patients. Cancers (Basel). (2024) 16:4008. doi: 10.3390/cancers16234008 39682194 PMC11640486

