# Correction: The intratumoral microbiome in colorectal cancer: origins, microenvironmental interactions, and new horizons in precision medicine

**DOI:** 10.3389/fimmu.2026.1991410

**Published:** 2026-09-29

**Authors:** Xuemei Li, Qian Wang, Qiang Yuan, Li Wang

**Affiliations:** 1 Department of Clinical Medicine, Chengdu Medical College, Chengdu, Sichuan, China; 2 Department of Gastroenterology, First Affiliated Hospital of Chengdu Medical College, Chengdu, Sichuan, China

**Keywords:** biomarkers, colorectal cancer, Fusobacterium nucleatum, immunotherapy, intratumoral microbiome, tumor microenvironment

There was a mistake in the **Generative AI statement** as published. The statement did not disclose the use of AI for figure editing. The corrected **Generative AI statement** appears below:

“The author(s) declared that generative AI was used in the creation of this manuscript. Some medical visual materials incorporated into **Figures 1**–**3** were generated with the assistance of Google Gemini (Nano Banana). The authors subsequently used BioRender to manually combine, edit, and arrange these materials together with other graphical elements to create the final figures. Generative AI was not used for the generation or analysis of research data, or for the interpretation of the study results.

**Figure 1:**
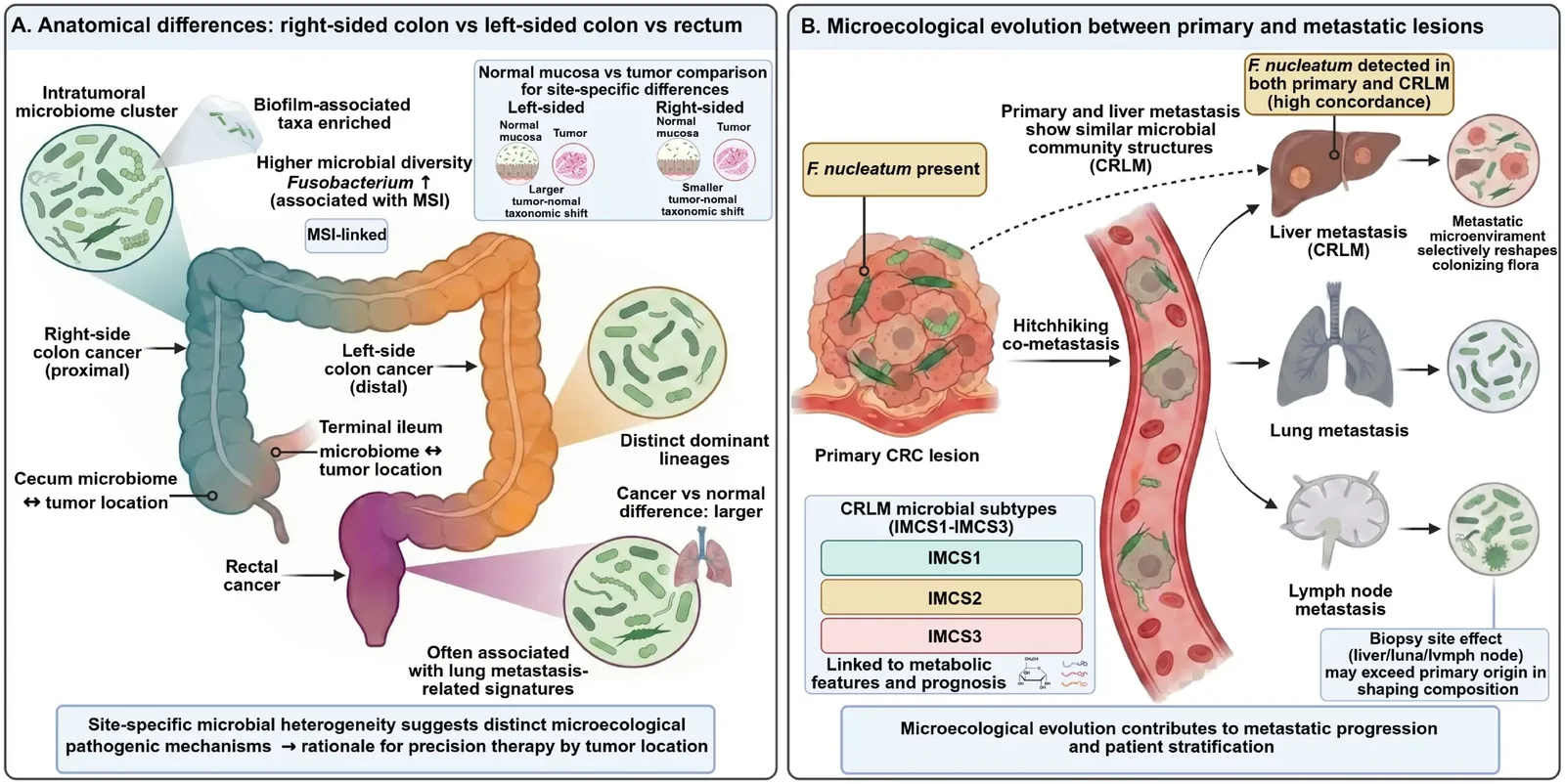
Compositional and distribution characteristics of the intratumoral microbiome in CRC. **(A)** Anatomical differences across CRC sites. Schematic comparison of intratumoral microbial lineages across right-sided (proximal) colon cancer, left-sided (distal) colon cancer, and rectal cancer. Right-sided colon tumors typically exhibit higher microbial diversity and are more prone to enrichment of biofilm-associated taxa; increased abundance of Fusobacterium is often associated with microsatellite instability (MSI). Left-sided colon cancer and rectal cancer display distinct dominant microbial lineages, and rectal cancer -associated microbial signatures are frequently linked to lung metastasis. The inset highlights site-dependent magnitudes of the “normal mucosa -tumor” shift, showing a larger tumor -normal difference in left-sided disease but a relatively smaller difference in right-sided disease. In addition, the cecal and terminal ileal microbiomes are associated with tumor location, indicating site-specific heterogeneity of the intratumoral microecology. **(B)** Microecological evolution between primary and metastatic lesions. Intratumoral microbes can accompany tumor cells during metastatic dissemination (“hitchhiking co-metastasis”), resulting in partial similarity of microbial community structures between primary tumors and metastatic lesions, particularly colorectal cancer liver metastasis (CRLM). Using Fusobacterium nucleatum as an example, high concordance can be observed between primary CRC and paired CRLM. Meanwhile, the metastatic microenvironment (liver, lung, lymph node) can selectively reshape the colonizing flora. The figure further illustrates that CRLM can be stratified into distinct subtypes based on intratumoral microbial community features (IMCS1 -IMCS3; intratumoral microbial community subtypes), which are associated with metabolic characteristics and prognosis, and emphasizes that in some settings the biopsy-site effect may outweigh the primary origin in shaping the microbial composition of metastatic lesions. Created in BioRender. Steven, T. (2026) https://BioRender.com/t4aum9z

**Figure 2:**
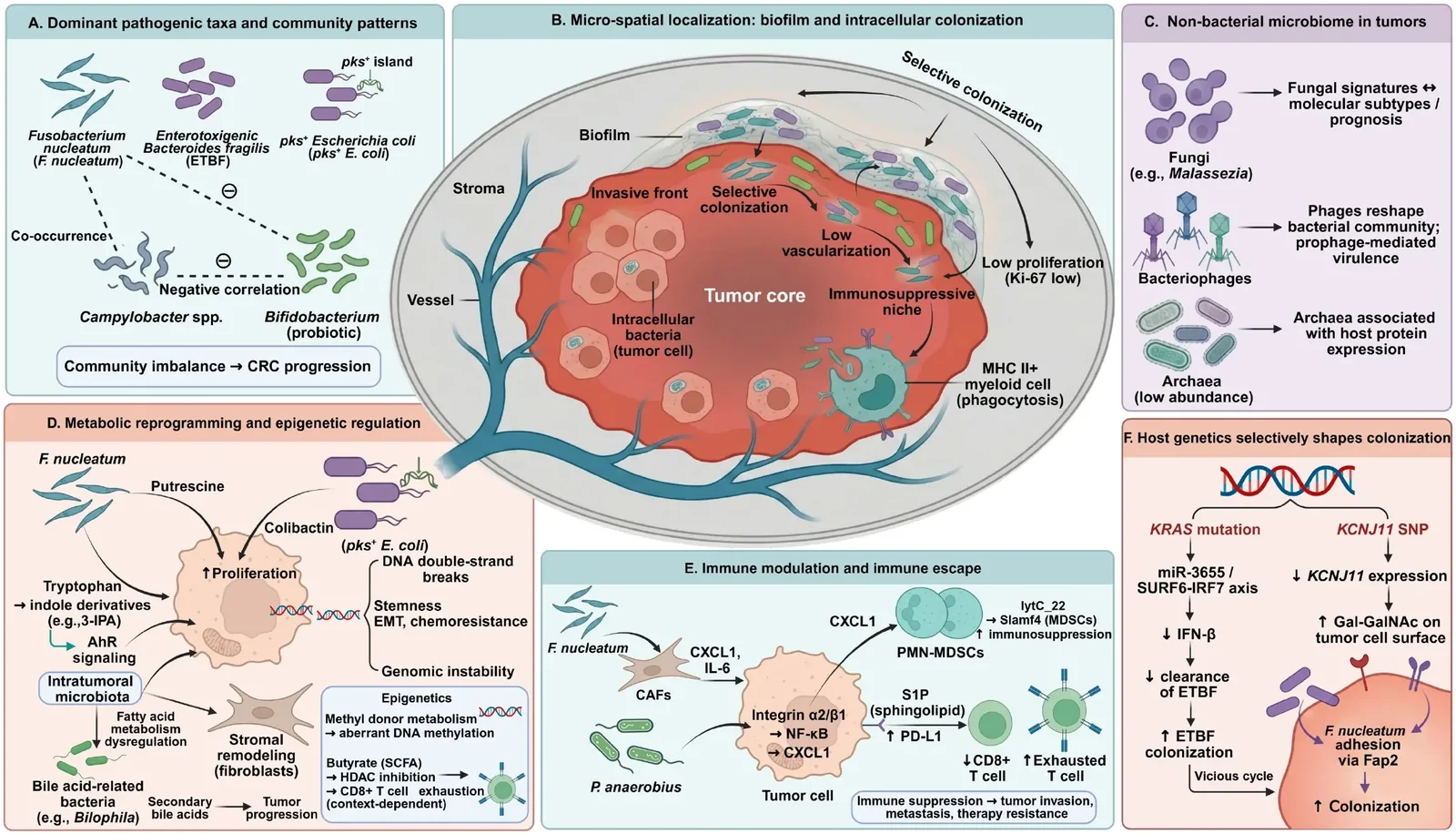
Core intratumoral microbiota and host -microbe interaction mechanisms in CRC. **(A)** Dominant pathogenic taxa and community patterns. Fusobacterium nucleatum (F. nucleatum), enterotoxigenic Bacteroides fragilis (ETBF), and pks island -harboring Escherichia coli (pks+ E. coli) represent core pro-tumorigenic taxa in CRC. F. nucleatum frequently co-occurs with other pathogens (e.g., Campylobacter spp.) and shows negative correlation with probiotics (e.g., Bifidobacterium), contributing to community imbalance and CRC progression. **(B)** Micro-spatial localization: biofilm and intracellular colonization. Intratumoral microbes display selective colonization, including biofilm formation at the invasive front and enrichment within microniches characterized by low vascularization, immunosuppressive features, and low proliferation (Ki-67 low). Microbes can localize intracellularly in tumor cells and be phagocytosed by major histocompatibility complex class II -positive (MHC II+) myeloid cells. **(C)** Non-bacterial microbiome in tumors. Fungi (e.g., Malassezia), bacteriophages, and archaea (low abundance) may associate with molecular subtypes/prognosis, reshape bacterial community structure, and contribute to host -microbe interactions. **(D)** Metabolic reprogramming and epigenetic regulation. F. nucleatum-derived metabolites (e.g., putrescine) promote tumor proliferation, while tryptophan-to-indole derivatives (e.g., 3-indolepropionic acid, 3-IPA) activate aryl hydrocarbon receptor (AhR) signaling to enhance stemness, epithelial -mesenchymal transition (EMT), and chemoresistance. pks+ (E) coli produces colibactin, inducing DNA double-strand breaks and genomic instability. Broader intratumoral microbiota perturbations drive fatty acid metabolism dysregulation and stromal remodeling (fibroblasts), and bile acid -related bacteria (e.g., Bilophila) contribute to secondary bile acids that support tumor progression. Epigenetically, altered methyl donor metabolism promotes aberrant DNA methylation, whereas butyrate, a short-chain fatty acid (SCFA), via histone deacetylase (HDAC) inhibition can induce context-dependent CD8+ T cell exhaustion. **(E)** Immune modulation and immune escape. F. nucleatum activates cancer-associated fibroblasts (CAFs) to secrete chemokine (C-X-C motif) ligand 1 (CXCL1) and interleukin-6 (IL-6), facilitating invasion and metastasis. Peptostreptococcus anaerobius triggers integrin α2/β1 -NF-κ?B signaling in tumor cells to induce CXCL1, recruiting polymorphonuclear myeloid-derived suppressor cells (PMN-MDSCs). The bacterial factor lytC_22 enhances MDSC immunosuppression via signaling lymphocytic activation molecule family member 4 (SLAMF4) on MDSCs. Microbial sphingolipid sphingosine-1-phosphate (S1P) upregulates programmed death-ligand 1 (PD-L1) on tumor cells, restricting effector T-cell expansion and increasing exhausted T cells, collectively driving immune suppression and therapy resistance. **(F)** Host genetics selectively shapes colonization. KRAS mutation -associated miR-3655/SURF6 -interferon regulatory factor 7 (IRF7) signaling reduces interferon beta (IFN-β), impairing ETBF clearance and increasing ETBF colonization in a vicious cycle. KCNJ11 single nucleotide polymorphism (SNP) -linked downregulation of KCNJ11 increases galactose -N-acetylgalactosamine (Gal -GalNAc) on tumor cells, promoting F. nucleatum adhesion via Fap2 and enhancing colonization. Created in BioRender. Steven, T. (2026) https://BioRender.com/bjwm3mm

**Figure 3:**
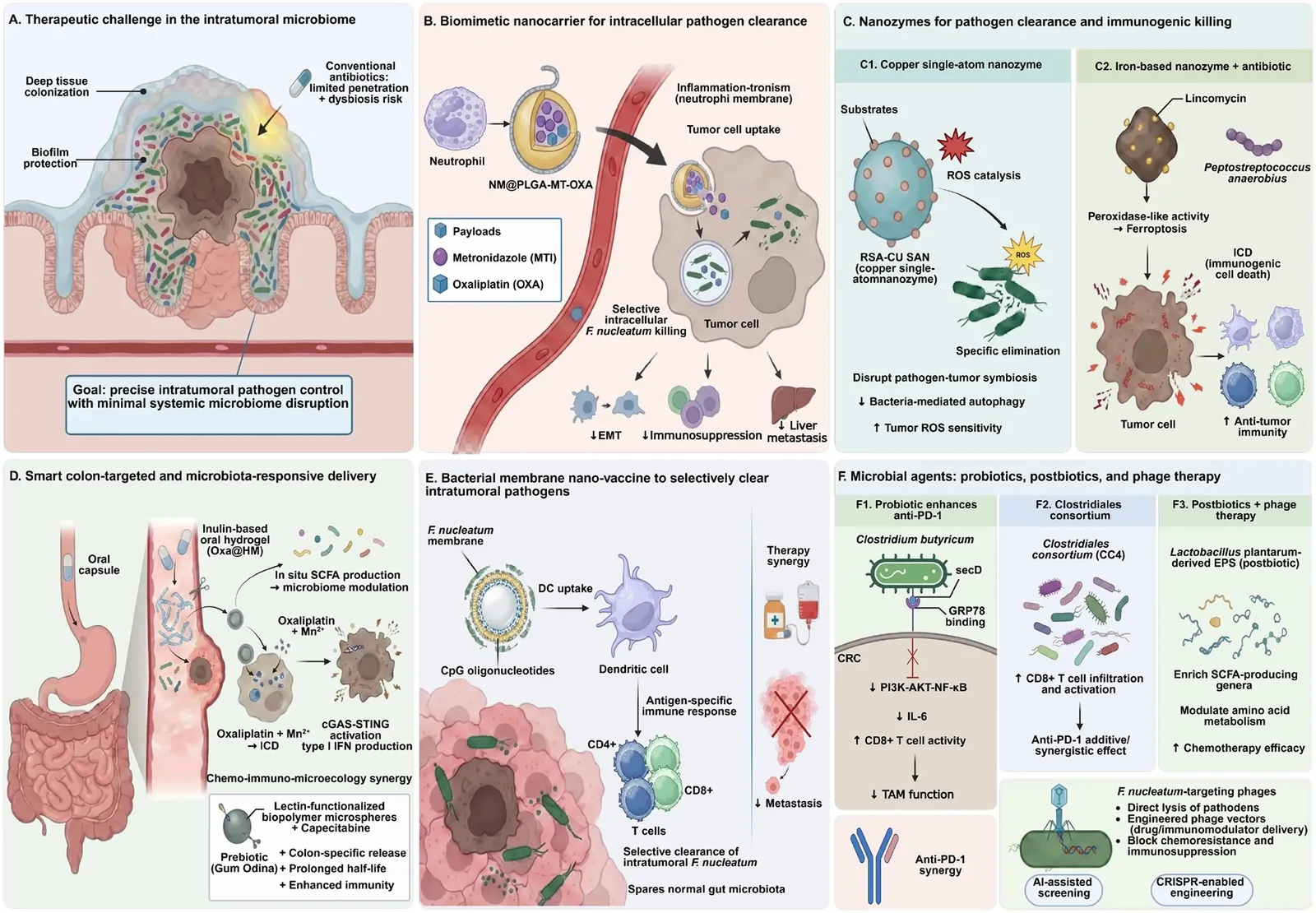
Therapeutic interventions targeting the intratumoral microbiome in CRC. **(A)** Therapeutic challenge in the intratumoral microbiome. Intratumoral microbes can colonize deep tumor tissues and are protected by biofilms, limiting antibiotic penetration and increasing the risk of systemic dysbiosis. The overarching goal is precise intratumoral pathogen control with minimal disruption of the host microbiome. **(B)** Biomimetic nanocarrier for intracellular pathogen clearance. Neutrophil membrane -coated poly (lactic-co-glycolic acid) nanoparticles co-loaded with metronidazole and oxaliplatin (NM@PLGA-MTI-OXA) exploit inflammation tropism to accumulate in tumors and be taken up by tumor cells, enabling selective killing of intracellular F. nucleatum. This strategy is depicted as reversing epithelial -mesenchymal transition (EMT) and reducing immunosuppression, thereby suppressing liver metastasis. **(C)** Nanozymes for pathogen clearance and immunogenic killing. (C1) A bovine serum albumin -supported copper single-atom nanozyme (BSA-Cu SAN) passively targets tumor sites and catalyzes reactive oxygen species (ROS) generation to specifically eliminate F. nucleatum, disrupt pathogen -tumor symbiosis, attenuate bacteria-mediated autophagy, and restore tumor sensitivity to ROS. (C2) A lincomycin-loaded iron-based nanozyme targets intratumoral Peptostreptococcus anaerobius; its peroxidase-like activity induces ferroptosis and triggers immunogenic cell death (ICD), promoting anti-tumor immunity. **(D)** Smart colon-targeted and microbiota-responsive delivery. An inulin-based oral hydrogel system (Oxa@HMI) is degraded by inulinase produced by colonic microbiota, releasing oxaliplatin-loaded hollow manganese oxide nanocarriers. This process supports in situ short-chain fatty acid (SCFA) production and microbiome modulation, while oxaliplatin and Mn²+ promote ICD and activate the cyclic GMP -AMP synthase -stimulator of interferon genes (cGAS -STING) pathway, achieving chemo -immuno -microecology synergy. A parallel strategy uses lectin-functionalized biopolymer microspheres loaded with capecitabine plus a prebiotic (Gum Odina) to enable colon-specific drug release, prolong half-life, and enhance anti-tumor immunity. **(E)** Bacterial membrane nano-vaccine to selectively clear intratumoral pathogens. A biomimetic nano-vaccine integrating F. nucleatum membranes with CpG oligodeoxynucleotides is taken up by dendritic cells (DCs), induces antigen-specific T-cell responses, and selectively clears intratumoral F. nucleatum while sparing normal gut microbiota, thereby improving chemotherapy efficacy and reducing metastasis. **(F)** Microbial agents: probiotics, postbiotics, and phage therapy. (F1) The probiotic Clostridium butyricum enhances anti-programmed cell death protein 1 (anti-PD-1) therapy via its surface protein secD binding to glucose-regulated protein 78 (GRP78), inhibiting phosphoinositide 3-kinase -protein kinase B -nuclear factor kappa-B (PI3K -AKT -NF-κB) signaling and decreasing interleukin-6 (IL-6), thereby increasing CD8+ T cell activity and weakening tumor-associated macrophage (TAM) function. (F2) A Clostridiales consortium (CC4) promotes intratumoral CD8+ T cell infiltration and activation and shows additive/synergistic effects with anti-PD-1. (F3) Lactobacillus plantarum -derived exopolysaccharides (EPS; postbiotic) enrich SCFA-producing genera and modulate amino-acid metabolism to enhance chemotherapy efficacy; engineered bacteriophages can directly lyse tumor-associated pathogens (including F. nucleatum) and serve as programmable vectors for drug/immunomodulator delivery, aided by artificial intelligence (AI) -assisted screening and clustered regularly interspaced short palindromic repeats (CRISPR) -enabled engineering. Created in BioRender. Steven, T. (2026) https://BioRender.com/eex0bgo

Any alternative text (alt text) provided alongside figures in this article has been generated by Frontiers with the support of artificial intelligence and reasonable efforts have been made to ensure accuracy, including review by the authors wherever possible. If you identify any issues, please contact us.”

There was a mistake in the caption of **Figure 1** as published. The captions did not include the BioRender license information. The corrected caption of **Figure 1** appears below.

“Compositional and distribution characteristics of the intratumoral microbiome in CRC. (A) Anatomical differences across CRC sites. Schematic comparison of intratumoral microbial lineages across right-sided (proximal) colon cancer, left-sided (distal) colon cancer, and rectal cancer. Right-sided colon tumors typically exhibit higher microbial diversity and are more prone to enrichment of biofilm-associated taxa; increased abundance of Fusobacterium is often associated with microsatellite instability (MSI). Left-sided colon cancer and rectal cancer display distinct dominant microbial lineages, and rectal cancer–associated microbial signatures are frequently linked to lung metastasis. The inset highlights site-dependent magnitudes of the “normal mucosa–tumor” shift, showing a larger tumor–normal difference in left-sided disease but a relatively smaller difference in right-sided disease. In addition, the cecal and terminal ileal microbiomes are associated with tumor location, indicating site-specific heterogeneity of the intratumoral microecology. (B) Microecological evolution between primary and metastatic lesions. Intratumoral microbes can accompany tumor cells during metastatic dissemination (“hitchhiking co-metastasis”), resulting in partial similarity of microbial community structures between primary tumors and metastatic lesions, particularly colorectal cancer liver metastasis (CRLM). Using Fusobacterium nucleatum as an example, high concordance can be observed between primary CRC and paired CRLM. Meanwhile, the metastatic microenvironment (liver, lung, lymph node) can selectively reshape the colonizing flora. The figure further illustrates that CRLM can be stratified into distinct subtypes based on intratumoral microbial community features (IMCS1–IMCS3; intratumoral microbial community subtypes), which are associated with metabolic characteristics and prognosis, and emphasizes that in some settings the biopsy-site effect may outweigh the primary origin in shaping the microbial composition of metastatic lesions. Created in BioRender. Steven, T. (2026) https://BioRender.com/t4aum9z”

There was a mistake in the caption of **Figure 2** as published. The captions did not include the BioRender license information. The corrected caption of **Figure 2** appears below.

“Core intratumoral microbiota and host–microbe interaction mechanisms in CRC. (A) Dominant pathogenic taxa and community patterns. Fusobacterium nucleatum (F. nucleatum), enterotoxigenic Bacteroides fragilis (ETBF), and pks island–harboring Escherichia coli (pks+ E. coli) represent core pro-tumorigenic taxa in CRC. F. nucleatum frequently co-occurs with other pathogens (e.g., Campylobacter spp.) and shows negative correlation with probiotics (e.g., Bifidobacterium), contributing to community imbalance and CRC progression. (B) Micro-spatial localization: biofilm and intracellular colonization. Intratumoral microbes display selective colonization, including biofilm formation at the invasive front and enrichment within microniches characterized by low vascularization, immunosuppressive features, and low proliferation (Ki-67 low). Microbes can localize intracellularly in tumor cells and be phagocytosed by major histocompatibility complex class II–positive (MHC II+) myeloid cells. (C) Non-bacterial microbiome in tumors. Fungi (e.g., Malassezia), bacteriophages, and archaea (low abundance) may associate with molecular subtypes/prognosis, reshape bacterial community structure, and contribute to host–microbe interactions. (D) Metabolic reprogramming and epigenetic regulation. F. nucleatum-derived metabolites (e.g., putrescine) promote tumor proliferation, while tryptophan-to-indole derivatives (e.g., 3-indolepropionic acid, 3-IPA) activate aryl hydrocarbon receptor (AhR) signaling to enhance stemness, epithelial–mesenchymal transition (EMT), and chemoresistance. pks+ (E) coli produces colibactin, inducing DNA double-strand breaks and genomic instability. Broader intratumoral microbiota perturbations drive fatty acid metabolism dysregulation and stromal remodeling (fibroblasts), and bile acid–related bacteria (e.g., Bilophila) contribute to secondary bile acids that support tumor progression. Epigenetically, altered methyl donor metabolism promotes aberrant DNA methylation, whereas butyrate, a short-chain fatty acid (SCFA), via histone deacetylase (HDAC) inhibition can induce context-dependent CD8+ T cell exhaustion. (E) Immune modulation and immune escape. F. nucleatum activates cancer-associated fibroblasts (CAFs) to secrete chemokine (C-X-C motif) ligand 1 (CXCL1) and interleukin-6 (IL-6), facilitating invasion and metastasis. Peptostreptococcus anaerobius triggers integrin α2/β1–NF-κB signaling in tumor cells to induce CXCL1, recruiting polymorphonuclear myeloid-derived suppressor cells (PMN-MDSCs). The bacterial factor lytC_22 enhances MDSC immunosuppression via signaling lymphocytic activation molecule family member 4 (SLAMF4) on MDSCs. Microbial sphingolipid sphingosine-1-phosphate (S1P) upregulates programmed death-ligand 1 (PD-L1) on tumor cells, restricting effector T-cell expansion and increasing exhausted T cells, collectively driving immune suppression and therapy resistance. (F) Host genetics selectively shapes colonization. KRAS mutation–associated miR-3655/SURF6–interferon regulatory factor 7 (IRF7) signaling reduces interferon beta (IFN-β), impairing ETBF clearance and increasing ETBF colonization in a vicious cycle. KCNJ11 single nucleotide polymorphism (SNP)–linked downregulation of KCNJ11 increases galactose–N-acetylgalactosamine (Gal–GalNAc) on tumor cells, promoting F. nucleatum adhesion via Fap2 and enhancing colonization. Created in BioRender. Steven, T. (2026) https://BioRender.com/bjwm3mm”

There was a mistake in the caption of **Figure 3** as published. The captions did not include the BioRender license information. The corrected caption of **Figure 3** appears below.

“Therapeutic interventions targeting the intratumoral microbiome in CRC. (A) Therapeutic challenge in the intratumoral microbiome. Intratumoral microbes can colonize deep tumor tissues and are protected by biofilms, limiting antibiotic penetration and increasing the risk of systemic dysbiosis. The overarching goal is precise intratumoral pathogen control with minimal disruption of the host microbiome. (B) Biomimetic nanocarrier for intracellular pathogen clearance. Neutrophil membrane–coated poly (lactic-co-glycolic acid) nanoparticles co-loaded with metronidazole and oxaliplatin (NM@PLGA-MTI-OXA) exploit inflammation tropism to accumulate in tumors and be taken up by tumor cells, enabling selective killing of intracellular F. nucleatum. This strategy is depicted as reversing epithelial–mesenchymal transition (EMT) and reducing immunosuppression, thereby suppressing liver metastasis. (C) Nanozymes for pathogen clearance and immunogenic killing. (C1) A bovine serum albumin–supported copper single-atom nanozyme (BSA-Cu SAN) passively targets tumor sites and catalyzes reactive oxygen species (ROS) generation to specifically eliminate F. nucleatum, disrupt pathogen–tumor symbiosis, attenuate bacteria-mediated autophagy, and restore tumor sensitivity to ROS. (C2) A lincomycin-loaded iron-based nanozyme targets intratumoral Peptostreptococcus anaerobius; its peroxidase-like activity induces ferroptosis and triggers immunogenic cell death (ICD), promoting anti-tumor immunity. (D) Smart colon-targeted and microbiota-responsive delivery. An inulin-based oral hydrogel system (Oxa@HMI) is degraded by inulinase produced by colonic microbiota, releasing oxaliplatin-loaded hollow manganese oxide nanocarriers. This process supports *in situ* short-chain fatty acid (SCFA) production and microbiome modulation, while oxaliplatin and Mn²+ promote ICD and activate the cyclic GMP–AMP synthase–stimulator of interferon genes (cGAS–STING) pathway, achieving chemo–immuno–microecology synergy. A parallel strategy uses lectin-functionalized biopolymer microspheres loaded with capecitabine plus a prebiotic (Gum Odina) to enable colon-specific drug release, prolong half-life, and enhance anti-tumor immunity. (E) Bacterial membrane nano-vaccine to selectively clear intratumoral pathogens. A biomimetic nano-vaccine integrating F. nucleatum membranes with CpG oligodeoxynucleotides is taken up by dendritic cells (DCs), induces antigen-specific T-cell responses, and selectively clears intratumoral F. nucleatum while sparing normal gut microbiota, thereby improving chemotherapy efficacy and reducing metastasis. (F) Microbial agents: probiotics, postbiotics, and phage therapy. (F1) The probiotic Clostridium butyricum enhances anti-programmed cell death protein 1 (anti-PD-1) therapy via its surface protein secD binding to glucose-regulated protein 78 (GRP78), inhibiting phosphoinositide 3-kinase–protein kinase B–nuclear factor kappa-B (PI3K–AKT–NF-κB) signaling and decreasing interleukin-6 (IL-6), thereby increasing CD8+ T cell activity and weakening tumor-associated macrophage (TAM) function. (F2) A Clostridiales consortium (CC4) promotes intratumoral CD8+ T cell infiltration and activation and shows additive/synergistic effects with anti-PD-1. (F3) Lactobacillus plantarum–derived exopolysaccharides (EPS; postbiotic) enrich SCFA-producing genera and modulate amino-acid metabolism to enhance chemotherapy efficacy; engineered bacteriophages can directly lyse tumor-associated pathogens (including F. nucleatum) and serve as programmable vectors for drug/immunomodulator delivery, aided by artificial intelligence (AI)–assisted screening and clustered regularly interspaced short palindromic repeats (CRISPR)–enabled engineering. Created in BioRender. Steven, T. (2026) https://BioRender.com/eex0bgo”

The original version of this article has been updated.

